# Photosystem II antenna complexes CP26 and CP29 are essential for nonphotochemical quenching in *Chlamydomonas reinhardtii*

**DOI:** 10.1111/pce.13680

**Published:** 2019-12-08

**Authors:** Stefano Cazzaniga, Minjae Kim, Francesco Bellamoli, Jooyoen Jeong, Sangmuk Lee, Federico Perozeni, Andrea Pompa, EonSeon Jin, Matteo Ballottari

**Affiliations:** ^1^ Dipartimento di Biotecnologie Università di Verona Verona Italy; ^2^ Department of Life Science Hanyang University Seoul South Korea; ^3^ Dipartimento di Scienze Biomolecolari Università degli Studi di Urbino Urbino Italy; ^4^ Istituto di Bioscienze e Biorisorse Consiglio Nazionale delle Ricerche Perugia Italy

**Keywords:** antenna complexes, Chlamydomonas, genome editing, microalgae, nonphotochemical quenching, photodamage, photoprotection, photosynthesis, photosystem, state transitions

## Abstract

Photosystems must balance between light harvesting to fuel the photosynthetic process for CO_2_ fixation and mitigating the risk of photodamage due to absorption of light energy in excess. Eukaryotic photosynthetic organisms evolved an array of pigment‐binding proteins called light harvesting complexes constituting the external antenna system in the photosystems, where both light harvesting and activation of photoprotective mechanisms occur. In this work, the balancing role of CP29 and CP26 photosystem II antenna subunits was investigated in *Chlamydomonas reinhardtii* using CRISPR‐Cas9 technology to obtain single and double mutants depleted of monomeric antennas. Absence of CP26 and CP29 impaired both photosynthetic efficiency and photoprotection: Excitation energy transfer from external antenna to reaction centre was reduced, and state transitions were completely impaired. Moreover, differently from higher plants, photosystem II monomeric antenna proteins resulted to be essential for photoprotective thermal dissipation of excitation energy by nonphotochemical quenching.

## Abstract 2

The functional role of the Photosystem II monomeric antenna complexes CP29 and CP26 was investigated in Chlamydomonas reinhardtii generating single and double mutants by CRISPR‐Cas9 genome editing demonstrating their key role in non‐photochemical quenching of both Photosystem I and II.

## 1 INTRODUCTION

Photosynthesis is the process that provides the continuous energy income needed to maintain life on our planet (Hill & Scarisbrick, 1940). The pigment‐binding complexes where light harvesting and primary photochemical reactions occur are named photosystems. In oxygenic photosynthesis, photosystem I (PSI) and photosystem II (PSII) are similarly organized with a core complex, where the primary photochemical reactions occur, surrounded by an array of antennae (Suga et al., 2015). Photosystems evolved their large array of antenna proteins in order to optimize light absorption within the visible region (Croce & van Amerongen, 2013; van Amerongen & Croce, 2013). However, when too much excitation energy is absorbed and the electron transport chain becomes saturated, photodamage may occur with formation of reactive oxygen species (Murchie & Niyogi, 2011). Within the photosynthetic machinery, due to the lower photosynthetic efficiency of PSII compared with PSI, PSII and its reaction centre have been indicated as the primary target of photoinhibition (Vass et al., 1992).

The peripheral antennae of PSII are in two different aggregation states: trimeric LHCII and monomeric CP29 (Lhcb4), CP26 (Lhcb5), and CP24 (Lhcb6), the latter found only in land plants (Alboresi, Gerotto, Giacometti, Bassi, & Morosinotto, 2010; Jansson, 1999). All antenna proteins are pigment‐binding proteins encoded by nuclear genes and share a similar structure, with three transmembrane domains and two amphipathic α‐helices (Matteo Ballottari, Girardon, Dall'Osto, & Bassi, 2012; Liu et al., 2004). In *Chlamydomonas reinhardtii*, PSII supercomplex structure has been observed by electron microscopy, revealing two layers of antenna proteins surrounding a homodimeric core complex (Tokutsu, Kato, Bui, Ishikawa, & Minagawa, 2012): The inner layer is composed of CP29, CP26, and S‐LHCII (S, strongly bound), forming with the core the C_2_S_2_ particle; the outer layer is made of M‐LHCIIs (moderately bound) forming, together with C_2_S_2_, the C_2_S_2_M_2_ complex. M‐LHCIIs are expected to be connected to the core through CP29. In *C. reinhardtii*, an additional LHCII (N‐LHCII) can be connected directly to the PSII core in the position occupied by CP24 in higher plants, forming the larger C_2_S_2_M_2_N_2_ complexes (Tokutsu et al., 2012). PSII antenna proteins are also involved in the State 1–State 2 transition (Allen, 1992), which regulates redistribution of excitation energy pressure between the two photosystems by disconnecting part of the LHCII trimers from PSII, which are then possibly transferred to PSI (Tokutsu, Iwai, & Minagawa, 2009).

Photosynthetic organisms have evolved different mechanisms to protect themselves from harmful excess light; the fastest of which is the thermal dissipation of excitation energy by nonphotochemical quenching (NPQ), a dissipative mechanism triggered by lumen acidification when the photosynthetic apparatus is saturated (Genty, Briantais, & Baker, 1989). In *C. reinhardtii*, the light harvesting complex stress‐related (LHCSR) subunits 1 and 3 are pigment‐binding LHC‐like proteins that sense luminal pH and trigger NPQ switching to a dissipative state (Girolomoni et al., 2019; Peers et al., 2009). In vascular plants, LHCSR is substituted by the PsbS subunit, which, however, does not bind pigments (Li et al., 2004). PsbS is also present in *C. reinhardtii*, but it is accumulated only transiently upon high‐light or ultraviolet exposure, and its contribution to NPQ is limited (Allorent et al., 2016; Correa‐Galvis et al., 2016; Tibiletti, Auroy, Peltier, & Caffarri, 2016). Both LHCSR proteins are overexpressed on prolonged high light treatment, but the molecular mechanisms at the base of NPQ and the interaction of LHCSR subunits with PSII supercomplexes are still not clear: LHCSR3 has been suggested to be bound to CP26 and/or to an LHCII trimer (Semchonok et al., 2017) suggesting a possible key role of monomeric subunits in the energy pathway from PSII to LHCSR subunits during NPQ induction. Moreover, it has been recently reported that phosphorylation of CP29 in *C. reinhardtii* is linked to LHCSR3 phosphorylation, being this process likely related to PSII assembly and repair in high light (Scholz et al., 2019). A knock‐out mutant without monomeric subunits was obtained in *Arabidopsis thaliana*, showing only a slower NPQ induction (Dall'Osto et al., 2017; Townsend et al., 2018). In microalgae, only RNA interference mutants of *C. reinhardtii* with individual monomeric subunits knocked down are reported in literature, revealing a peculiar role of CP29 in state transitions (Tokutsu et al., 2009), but the role on CP26 and CP29 in NPQ has never been investigated yet.

In this paper, we present the characterization of a *C. reinhardtii* double mutant, obtained with CRISPR‐Cas9 technology, completely depleted of CP26 and CP29 subunits. This mutant presents reduced photosynthetic efficiency and impaired state transitions. Surprisingly, the lack of CP26 and CP29 completely abolishes NPQ induction even in the presence of LHCSR1 and LHCSR3 subunits, implying that LHCSR needs the interaction with monomeric PSII antenna in order to perform its quenching function.

## 2 MATERIALS AND METHODS

### 2.1 Strains and culture conditions

*C. reinhardtii* wild type (Wt; CC503) and mutant strains were grown at 24°C in high‐salts (HS) medium (Harris & Harris, 2008) on a rotary shaker in Erlenmeyer flasks under continuous illumination with white LED light at 100‐μmol photons m^−2^ s^−1^. High light acclimation was induced by growing cells for 2 weeks at 500‐μmol photons m^−2^ s^−1^ in HS.

### 2.2 Generation of *C. reinhardtii* mutants by CRISPR‐Cas9 genome editing

Delivery of the DNA‐free CRISPR‐Cas9 RNP complex into cell was performed according to Baek et al. (2016) and Shin et al. (2019) with a few modifications. In brief, Cas9 protein (100 μg; ToolGen, South Korea) and in vitro transcribed sgRNA (70 μg), which was synthesized by using GeneArt™ Precision gRNA Synthesis Kit following the manufacturer's protocol (ThermoFisher, MA, USA), were mixed to form the RNP complex. The premixed RNP complex with and/or without *aph7* DNA cassette was introduced to the cells by Biorad Gene Pulser Xcell™ electroporation system (the *aph7* gene was prepared by PCR amplification from pChlamy3 vector with specific primer sets [F: ATGATTCCGCTCCGTGTAAATG, R: AGTACCATCAACTGACGTTACATTC]). After CRISPR‐Cas9 transformation, cells were incubated in TAP liquid medium supplemented with 40‐mM sucrose for 12 hr and harvested for genotype characterization or immediately diluted (2 × 10^3^ cells) and plated on TAP medium containing 1.5% agar to obtain single colonies. In the case of *k9* mutant strains, the colonies were screened by Fv/Fm fluorescence signal using a Walz Imaging PAM System (M‐series). For the selection of CRISPR‐Cas9–mediated antibiotic resistance knock‐in–derived mutants, cells were plated on TAP medium with hygromycin (25 μg/ml). Putative mutants screened were further analysed by Sanger sequencing to confirm the indel mutations or antibiotic resistance insertion. In the case of knock‐in mutants, single insertion events were confirmed by southern blot analysis.

### 2.3 Isolation of genomic DNA and complementary DNA preparation and quantitative reverse transcription PCR

Genomic DNA was extracted from *C. reinhardtii* using a protocol modified from Tanksley et al. (1992). Genomic DNA (20 μg) was digested with different restriction enzymes (NcoI and KpnI, Takara, Kusatsu, Japan). The fragments were separated on a 0.8% agarose gel, transferred onto a positively charged nylon membrane (Hybond‐N+, GE Healthcare, Chicago, IL, USA), and cross‐linked using shortwave ultraviolet light (254 nm). The probe was designed to cover 467 bp of the hygromycin resistance gene in pChlamy3 vector and was amplified from genomic DNA by PCR with the forward primer 5′‐ATGATTCCTACGCGAGCCTG‐3′ and reverse primer 5′‐ATCCGGCTCATCACCAGGTA‐3′. The amplified probe was labelled with alkaline phosphatase using the Gene Images AlkPhos Direct Labeling and Detection System kit (GE Healthcare, Chicago, IL, USA). Labelling, hybridization, washing, and signal detection were conducted according to the manufacturer's protocol. Total RNA was extracted from cells in the exponential phase with RNeasy Plant Mini Kit (Qiagen, Hilden, Germany). Complementary DNA (cDNA) was synthesized from total RNA by using 2X reverse transcription master premix (ELPiS Biotech, Daejeon, Korea) and amplified using SYBR premix (Takara, Kusatsu, Japan) in a Thermal Cycler Dice Real Time System TP 8200 (Takara, Kusatsu, Japan). The gene for the “receptor for activated C kinase 1 (RACK1)” was used as a reference and was amplified with the forward primer 5′‐GGCTGGGACAAGATGGTCAA‐3′ and reverse primer 5′‐GAGAAGCACAGGCAGTGGAT‐3′.

### 2.4 Membrane preparation and fractionation

Thylakoid membranes were isolated as previously described (Bonente et al., 2008). Fractionation of pigment–protein complexes was performed starting from thylakoid membranes concentrated at 0.5 mg/ml of Chls, solubilized with 0.8% α‐DM and 10‐mM HEPES, pH 7.8. Solubilized samples were then fractionated by ultracentrifugation in a 0.1‐ to 1‐M sucrose gradient containing 0.06% α‐DM and 10‐mM HEPES, pH 7.8 (22 hr at 280,000 g, 4°C).

### 2.5 SDS‐PAGE electrophoresis and immunoblotting

SDS‐PAGE analysis was performed using the Tris‐Tricine buffer system (Schägger & von Jagow, 1987), followed by Coomassie blue staining. For immunotitration, thylakoid samples were loaded for each sample and electroblotted on nitrocellulose membranes; then, proteins were quantified with an alkaline phosphatase–conjugated antibody system. αCP26 (AS09 407**)**, αCP29 (AS04 045), αCP43 (AS11 1787), αPSAA (AS06 172), αLHCSR1 (AS14 2819), αLHCSR3 (AS14 2766), and αSTT7 (AS15 3080); antibodies were purchased from Agrisera.

### 2.6 Pigment analysis

Chlorophyll and carotenoids content were analysed by high‐performance liquid chromatography upon pigment extraction in 80% acetone as described in Lagarde, Beuf, and Vermaas (2000).

### 2.7 Spectroscopy and fluorescence

Absorption spectra of pigments, extracted from intact cells using 80% acetone buffered with Na_2_CO_3_, were measured with Jasco V‐550 ultaviolet‐visible spectrophotometer. Spectra were fitted as previously described with different pigment absorption spectra (Croce, Canino, Ros, & Bassi, 2002). Low‐temperature fluorescence spectra were performed on frozen samples with BeamBio custom device equipped with USB2000+ spectrometer (OceanOptics) and 475‐nm LED light sources for excitation.

### 2.8 Photosynthetic parameters and NPQ measurements

Photosynthetic parameters ΦPSII, qL, electron transport rate (ETR), and NPQ were obtained by measuring with a DUAL‐PAM‐100 fluorimeter (Heinz–Walz) chlorophyll fluorescence of intact cells, at room temperature in a 1 × 1‐cm cuvette mixed by magnetic stirring. ΦPSII, qL, and ETR were measured and calculated according to Baker (2008) and Van Kooten and Snel (1990) at steady‐state photosynthesis upon 20 min of illumination. NPQ measurements were performed on dark‐adapted intact cells, with a saturating light of 4,000‐μmol photons m^−2^ s^−1^ and actinic lights from 150‐ to 3,900‐μmol photons m^−2^ s^−1^. PSII functional antenna size was measured from fast chlorophyll induction kinetics induced with a red light of 11‐μmol photons m^−2^ s^−1^ on dark‐adapted cells (~2·10^6^ cells/ml) incubated with 50‐μM DCMU (Malkin, Armond, Mooney, & Fork, 1981). The reciprocal of time corresponding to two thirds of the fluorescence rise (*τ*_2/3_) was taken as a measure of the PSII functional antenna size (Malkin et al., 1981). Proton motive force upon exposure to different light intensities was measured by electrochromic shift (ECS) with MultispeQ V2.0 (PhotosynQ) according to Kuhlgert et al. (2016). State transitions were induced with the protocol present in Drop, Yadav, Boekema, and Croce (2014). State transitions were estimated as (Fm_PSIS2_ − Fm_PSIS1_)/Fm_PSIS2_ where Fm_PSIS1_ and Fm_PSIS2_ are the maximal PSI fluorescence in State 1 and State 2, respectively. The oxygen evolution activity of the cultures was measured at 25°C with a Clark‐type O_2_ electrode (Hansatech), during illumination with light from a halogen lamp (Schott). Measurements were performed in 1‐ml cell suspension concentrated at 5 × 10^6^ cell/ml.

### 2.9 77‐K fluorescence

Low‐temperature quenching measures were performed according to Girolomoni et al. (2019). Cells were frozen in liquid nitrogen after being dark adapted or right after 10 min of illumination at 1,200‐μmol photons m^−2^ s^−1^, and fluorescence emission spectra were recorded. Green fluorescent protein was added to all samples as an internal standard for fluorescence emission spectra normalization.

### 2.10 Statistics analysis

All the experiments herein described were performed at least on two independent lines for each mutant genotype in at least four independent biological experiments. Statistical analysis was performed by using two‐sided Student's *t* test.

### 2.11 Data and strains availability

The authors declare that the data supporting the findings of this study are available within the paper and its supplementary information files. All the strains described in this work are fully available upon request to the corresponding author.

## 3 RESULTS

### 3.1 Generation of *C. reinhardtii* mutants on monomeric CP26 and CP29 subunits

**Figure 1:**
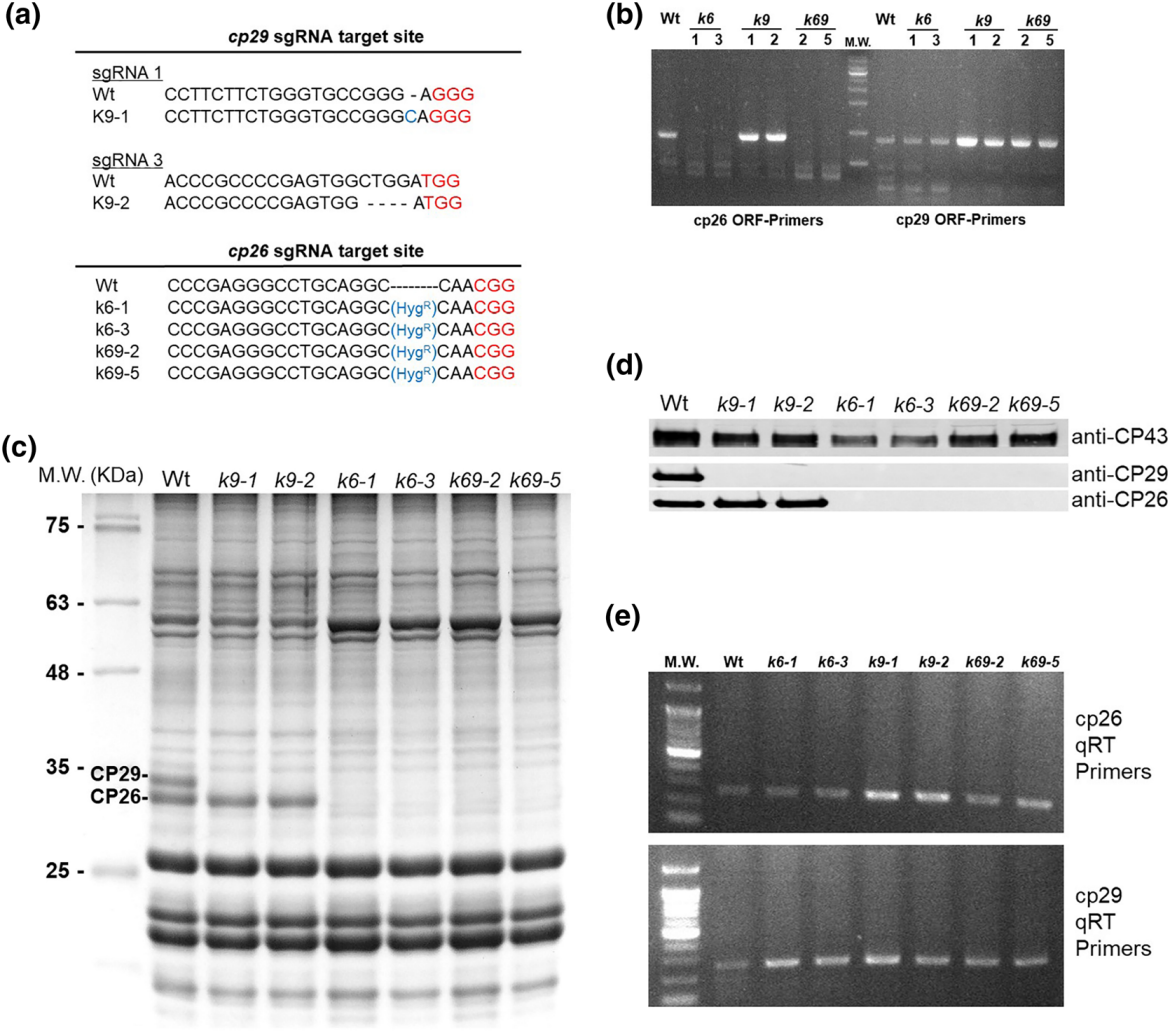
CP26‐ and CP29‐targeted gene disruption in *Chlamydomonas reinhardtii* by CRISPR/Cas9 genome editing. (a) DNA sequence alignment of wild type (Wt) and mutants on cp26 (*k6*) and cp29 (*k9*) obtained by CRISPR/Cas9 genome editing. The 20‐bp target sequence of sgRNA is reported before the red‐coloured PAM sequence. In the case of *k9* mutant, insertions (*k9‐1*) or deletions were induced (*k9‐2*) by non‐homologous repair, whereas in the case of *k6* mutant, a Cas9‐mediated hygromycin resistance cassette insertion was obtained in the target site. Double‐mutant *k69* was obtained by hygromycin resistance cassette insertion in a *k9* background. (b) PCR amplification of *cp26* and *cp29* CDS sequences from Wt, *k6*, *k9*, and *k69* cDNA. In the case of *k6* and *k69* mutants, the *cp26* CDS cannot be amplified due to hygromycin cassette insertion. (c) SDS‐PAGE analysis of Wt and mutant strains performed with the Tris‐Tricine buffer system. Fifteen microgram of Chl was loaded in each lane. Molecular weight (MW) markers are indicated on the left. CP26 and CP29 bands are marked. (d) Immunoblot with specific antibodies directed against CP26 and CP29 on the same lanes of (c). Immunoblot against CP43 was added as control of the loading. (e) qRT‐PCR on *cp26* and *cp29* genes in Wt and mutant strains. *rack1* gene was used as control for qRT‐PCR (see details in Figure S3)

Monomeric PSII antenna mutants in *C. reinhardtii* were generated using CRISPR‐Cas9 technology (Baek et al., 2016). First, the *cp29* gene (*Cre17.g720250*) was mutated by non‐homologous end joining: Two different sgRNA targets gave successful results, leading to introduction of insertions or deletions (Figures 1 and S1). The two selected mutant lines were named *k9‐1* and *k9‐2* (knock‐out CP29 mutants).

Thylakoid membrane polypeptides isolated from Wt and *k9* were separated on a denaturing gel (Figure 1). *k9* mutants lacked the band corresponding to CP29, and the complete absence of the protein was confirmed by western blot analysis using a specific antibody (Figure 1d). The *cp26* gene was subsequently targeted in Wt and *k9.2* strains: Two lines selected (for hygromycin resistance) from Wt background were named *k6* mutants (for knock‐out CP26), whereas the lines obtained from *k9.2* background were named *k69* (for knock‐out CP26 CP29). SDS‐PAGE and western blot analysis confirmed that both *k6* and *k69* mutants were completely depleted of CP26 (Figure 1). Surprisingly, both *k6* and *k69* lines also lacked the CP29 protein even if the gene was not targeted in *k6* mutant strains (Figures 1 and S2). In order to investigate if the absence of CP29 in *k6* mutant was related to a transcriptional or post‐transcriptional event, transcription profiles of *lhcb4* and *lhcb5* genes were investigated in Wt and mutant lines. As reported in Figures 1e and S3, the transcription of *cp29* and *cp26* genes was not impaired in *k6*, *k9*, and *k69* mutants compared with Wt, suggesting that the absence of CP26 and CP29 accumulation was related to translation or post‐translation events. Because *k6* and *k69* showed the same pattern of monomeric subunits and phenotype, the following characterization was focused on *k9* and *k69* strains.

### 3.2 Pigments composition and stoichiometry of pigment–protein complexes

When grown in HS medium (Harris & Harris, 2008) at 100 μmol m^−2^ s^−1^, *k9* and *k69* showed a slight but not significant reduction of chlorophyll (Chl) content compared with Wt; the Chl/carotenoid (Car) ratio was also similar in all the three genotypes (Table 1). Car composition determined by high‐performance liquid chromatography analysis was similar in the three genotypes with an increase of neoxanthin and violaxanthin and a decrease of β‐carotene in the *k69* mutant (Table 1). In these growth conditions, no zeaxanthin accumulation was detected in any genotype.

**Table 1:** Pigment content, Fv/Fm, and PSII antenna size

						Car/100 Chl				
	Chl, pg/cell	Chl a/b	Chl/Car	Fv/Fm	PSII Antenna Size, au	Neo	Viola	Anth	Lute	β‐Car
Wt	1.55 ± 0.09	2.68 ± 0.03	2.92 ± 0.17	0.67 ± 0.07	100 ± 8	4.92 ± 0.05	4.79 ± 0.28	0.75 ± 0.35	15.26 ± 0.48	8.52 ± 0.10
*k9*	1.54 ± 0.17	2.34 ± 0.01*	2.91 ± 0.11	0.51 ± 0.09*	144 ± 25*	4.95 ± 0.01	5.43 ± 0.02*	0.61 ± 0.01	14.89 ± 0.02	8.49 ± 0.02
*k69*	1.41 ± 0.14	1.92 ± 0.10* ^,^ **	3.05 ± 0.06	0.43 ± 0.08*	258 ± 89* ^,^ **	5.83 ± 0.26* ^,^ **	6.56 ± 0.80* ^,^ **	0.29 ± 0.09* ^,^ **	14.00 ± 0.45* ^,^ **	6.11 ± 0.57* ^,^ **

*Note*. All data were collected from cells grown in high salts at 100‐μmol photons m^−2^ s^−1^. PSII antenna size data are normalized to 1/*τ*_2/3_ value of wild type (Wt). Single carotenoid values are normalized to 100 chlorophylls. Data are expressed as mean ± *SD* (*n* = 4).

Abbreviations: β‐Car, β‐Carotene; Anth, antheraxanthin; Car, carotenoids; Chl, chlorophylls; Lute, lutein; Neo, neoxanthin; Viola, violaxanthin; Zea, zeaxanthin.

* Values that are significantly different (Student's *t* test, *P* < 0.05) from the Wt.

** Data that are significantly different between *k6* and *k69*.

**Figure 2:**
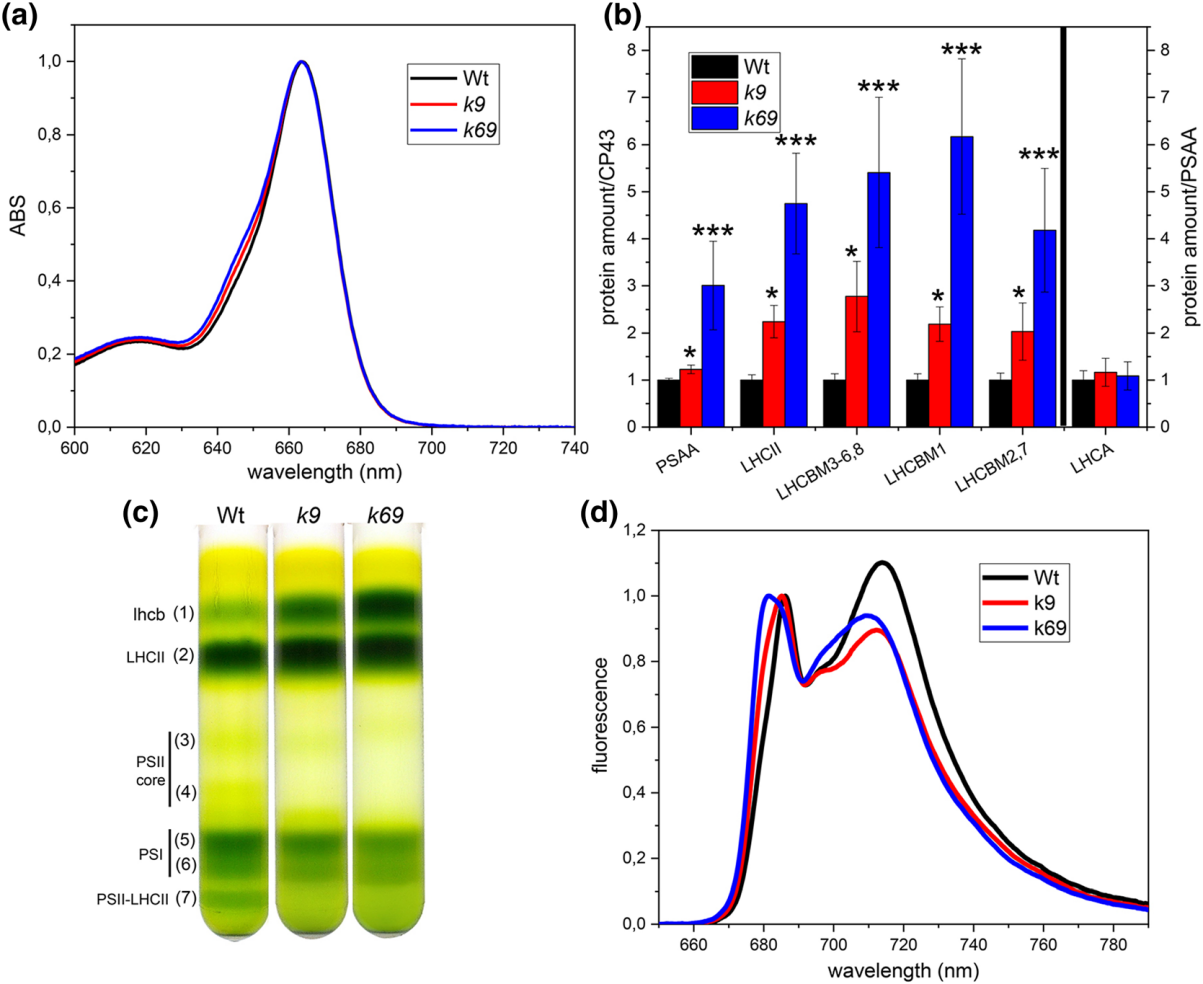
Organization of thylakoid membranes. (a) Absorption (ABS) spectra of acetone‐extracted pigments from wild‐type (Wt, black), *k9* (red), and *k69* (blue) cells. Spectra are normalized to the maximum of absorbance at 663 nm (Chl a absorption). The contribution of Chl b to absorption is visible at ~650 nm. (b) Immunotitration of thylakoid proteins using specific antibodies against PSAA, LHCII, and LHCA. Data were corrected for CP43 amount and normalized to Wt ratio. LHCA was corrected for PSAA amount. LHCII is shown as total amount (LHCII) or divided for three bands that are separated on the Tris‐Tricine buffer system (see Figure S2). Data are expressed as mean ± standard deviation (*n* = 4). ^*^Values that are significantly different (Student's *t* test, *P* < 0.05) from the WT. ^**^Data that are significantly different between *k6* and *k69*. (c) Sucrose density gradient fractionation of WT, *k9*, and *k69* solubilized with 0.8% dodecyl‐α‐d‐maltoside (α‐dM). Composition of the green bands is indicated on the left according to Semchonok et al. (2017); Wt in black, *k9* in grey, and *k69* in light grey. (d) Low‐temperature fluorescence of Wt, *k9*, and *k69* cells excited at 475 nm. Emission spectra are normalized at the maximum emission on PSII peak. All data were collected from algae grown in high salts at 100‐μmol photons m^−2^ s^−1^

The Chl a/b ratio was remarkably affected by the absence of monomeric subunits, being reduced in *k9* and even further decreased in *k69* in comparison with Wt (Figure 2a and Table 1), indicating a possible increment of PSII antenna proteins. In order to confirm this, we determined the relative amount of protein components of the photosynthetic apparatus by immunoblotting on LHCII, LHCI, CP43 (subunit of PSII core), and PSAA (subunit of PSI core; Figures 2b and S4; Ballottari, Dall'Osto, Morosinotto, & Bassi, 2007). Deletion of monomeric subunits had a strong effect on both PSII/PSI and LHCII/PSII ratios: In the *k9* mutant, there was a relative PSI increment of ~23% with respect to Wt, whereas in the double‐mutant *k69*, the amount of PSI was more than doubled. The effect of CP26 and CP29 mutations on LHCII/PSII was even stronger: In *k9*, the LHCII amount was doubled, whereas the *k69* mutants showed an approximately fivefold increase. The absence of CP26 and CP29 did not affect the PSI antenna because the LHCI/PSI ratio was the same as the Wt (Figures 2b and S4).

The organization of pigment‐binding complexes was further analysed by sucrose gradient upon solubilization of thylakoid membranes with dodecyl‐α‐d‐maltoside (α‐dM; Figure 2c). Different green bands were resolved; from top to bottom, the bands corresponded to LHC protein in monomeric state (Fraction 1), trimeric LHCII (Fraction 2), PSII core complex (Fractions 3 and 4), PSI‐LHCI (Fractions 5 and 6), and PSII‐LHCII supercomplex (Fraction 7; Drop et al., 2014). In Wt, the upper band contained CP29, CP26, and LHCII, monomerized by solubilization, whereas in *k69*, the band contained only the latter. The densitometry analysis of the band of the gradient confirmed the data from the immunotitration (Figure S5) with an increment of the bands corresponding to antenna proteins (Fractions 1 + 2) and a decrease of those corresponding to PSII core (Fractions 5 + 6). The bands corresponding to PSII‐LHCII supercomplex were absent in *k9* and *k69*, indicating that in mutants, these complexes were not assembled or they were easily dissociated by solubilization.

The functional antenna size of PSII in Wt and mutant strains was then determined by measuring fast chlorophyll florescence induction in the presence of DCMU (Malkin et al., 1981), being inversely proportional to the rise time. Mutants showed an antenna size increment of ~1.5‐fold in *k9* and 2.5‐fold in the *k69* double mutant with respect to Wt (Table 1 and Figure S6), consistently with the increased LHCII content and decreased Chl a/b ratio measured in these strains.

### 3.3 Photosynthetic efficiency and state transition

**Figure 3:**
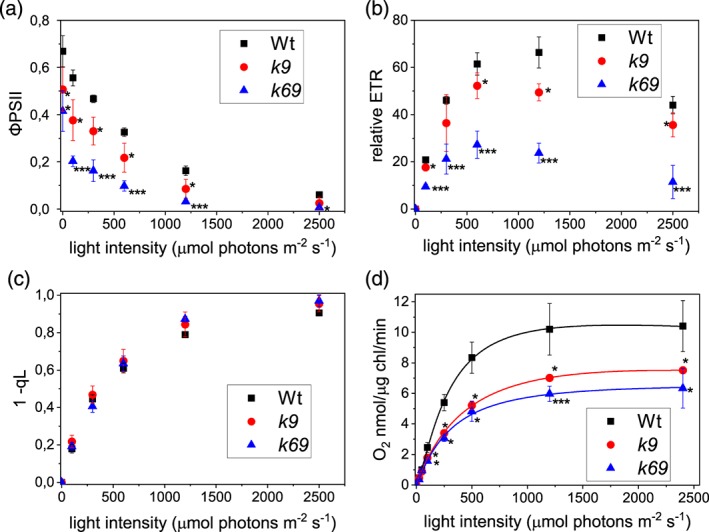
Characterization of the photosynthetic electron flow. Dependence of the (a) PSII operating efficiency (ΦPSII), (b) relative electron transport rate (ETR), (c) 1‐qL (estimates the fraction of PSII centres with reduced Q_A_), and (d) photosynthetic O_2_ evolution on actinic light intensity for wild type (Wt, black), *k9* (red), and *k69* (blue). Net photosynthetic rate data were fitted with Hill equation. Data are expressed as mean ± *SD*. *n* > 3. All data were collected from algae grown in high salts at 100‐μmol photons m^−2^ s^−1^. ^*^Values that are significantly different (Student's *t* test, *P* < 0.05) from Wt. ^**^Data that are significantly different between *k6* and *k69*

Fluorescence induction analysis in dark‐adapted cells (Butler, 1973) revealed a significant decrease of PSII (Fv/Fm) in mutants compared with Wt (Table 1), suggesting that energy transfer from LHCII to PSII reaction centre was less efficient in the absence of monomeric subunits. In particular, on a Chl basis, a clear increase of F_0_ was evident in the mutant strains (Figure S8). Accordingly, 77‐K fluorescence emission spectra (Figure 2d) revealed a blue shift in the first peak from 686 nm in Wt to 681 in *k69* mutant, with a 680‐nm shoulder evident also in *k9* mutant: This 681‐nm peak corresponds to light harvesting antennae unable to transmit their energy to PSII (Garnier, Maroc, & Guyon, 1986). In order to evaluate how this defective connection affects photosynthetic complexes, microalgae were illuminated at different light intensities, and PSII operating efficiency (ΦPSII), ETR, and photochemical quenching (1‐qL) were recorded (Figure 3a–c). Double‐mutant *k69* showed a reduction of ΦPSII and ETR at all the lights tested; the single‐mutant *k9* showed a reduction of these parameters with readings comprised between the Wt and the double‐mutant values. The Q_A_ redox state of the three genotypes was similar after 20 min of illumination at all the lights tested. To further investigate the functional properties of the photosynthetic machinery in the monomeric antenna mutants, the light‐saturation curve of photosynthesis, monitored as oxygen evolution, was measured on Wt and mutant strains (Figure 3d). The rate of oxygen release was lower in the two mutants compared with Wt at all light intensities tested, and the slope of light saturation curve initial linear increase was reduced (Table S1). These data confirmed that the quantum yield of photosynthetic apparatus is impaired in these genotypes, especially in double‐mutant *k69*. Dark respiration was instead not affected by the absence of CP26 and CP29.

**Figure 4:**
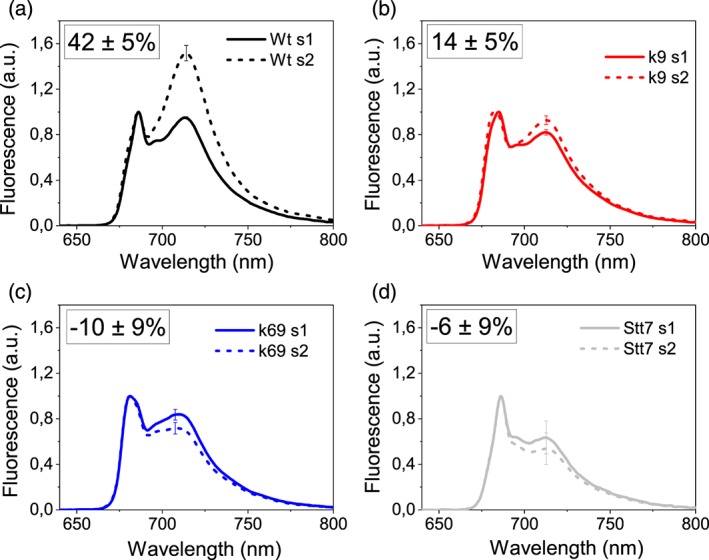
State transitions. Low‐temperature fluorescence emission spectra of the cells of (a) wild type (Wt), (b) *k9*, (c) *k69*, and (d) *stt7* after induction of State 1 (s1, solid line) or State 2 (s2, dashed line). The excitation wavelength was 475 nm, and spectra are normalized to the maximum in PSII emission. Error bars are indicated on PSI maximum emission (*n* = 4). The inset shows the estimation of state transition (see Section 2 for details)

The roles of CP26 and CP29 in state transitions were then investigated by inducing State 1 and State 2 as previously described (Drop, Yadav K N, et al., 2014) using the *stt7* mutant, unable to perform state transitions, as control (Depege, Bellafiore, & Rochaix, 2003). In the Wt in State 1, fluorescence on PSII (maximum at 685 nm) and PSI (maximum at 714 nm) had similar intensities; when State 2 was induced, the fluorescence on PSI increased compared with the PSII value (Figure 4). The *stt7* mutant was not able to increase the PSI fluorescence in State 2. PSI fluorescence change in the two states in Wt was 42 ± 12%: *k69* double mutant was unable to perform state transitions as in the case of the negative control, *stt7* mutant. Single‐mutant *k9* retained a small fraction of state transitions, but the mechanism was hampered (14 ± 5%) compared with Wt. The defects in state transitions observed in the *k9* and *k69* mutant strains (Figure 4) were not related to a different accumulation of the phosphorylating enzyme STT7, which was similarly accumulated in mutant strains compared with Wt (Figure S7). The treatment inducing State 2 in Wt caused in *k9* mutant a shift of PSII emission from 686 to 681 nm (Figure 4), indicating that in *k9* mutant, when state transitions are induced, LHCII detaches from PSII complex but only a part of it is able to shift to PSI. In State 2, the shape of PSII emission was identical to *k69* double mutant with a peak at 681 nm, suggesting that most of the LHCII trimers were already disconnected from PSII in both States 1 and 2.

### 3.4 Non‐photochemical quenching

The specific role of different PSII antenna in NPQ mechanism has long been debated (Ahn et al., 2008; Dall'Osto et al., 2017; Ruban et al., 2007; Townsend et al., 2018). We thus investigated the ability of Wt, *k9*, and *k69* to undergo quenching of Chl fluorescence upon exposure to excess light. *C. reinhardtii* needs to be adapted to high light conditions to fully activate NPQ mechanism; for this reason, Wt and mutant strains were grown for 2 weeks at 500‐μmol photons m^−2^ s^−1^ in autotrophy. After adaptation to high light, reduced Chl/cell and Chl/Car ratios were evident with no significant difference between Wt and mutant strains (Table S2). In the Wt, the Chl a/b ratio was similar in the two growth conditions (2.68 in low light vs. 2.70 in high light), although in *k9* and *k69*, a notable increase of Chl a/b ratio was measured: 1.92 in low light versus 2.48 in high light for *k69* and 2.55 versus 2.34 for *k9*. This indicates that *k9* and *k69* mutants, in conditions of excess light, decrease their antenna content to a level similar to Wt. As reported in Table S3, the Fv/Fm values of high light acclimated Wt and mutant strain were consistent with the values observed in low light acclimated ones, demonstrating the presence of active PSII even when samples were acclimated to high irradiance.

**Figure 5:**
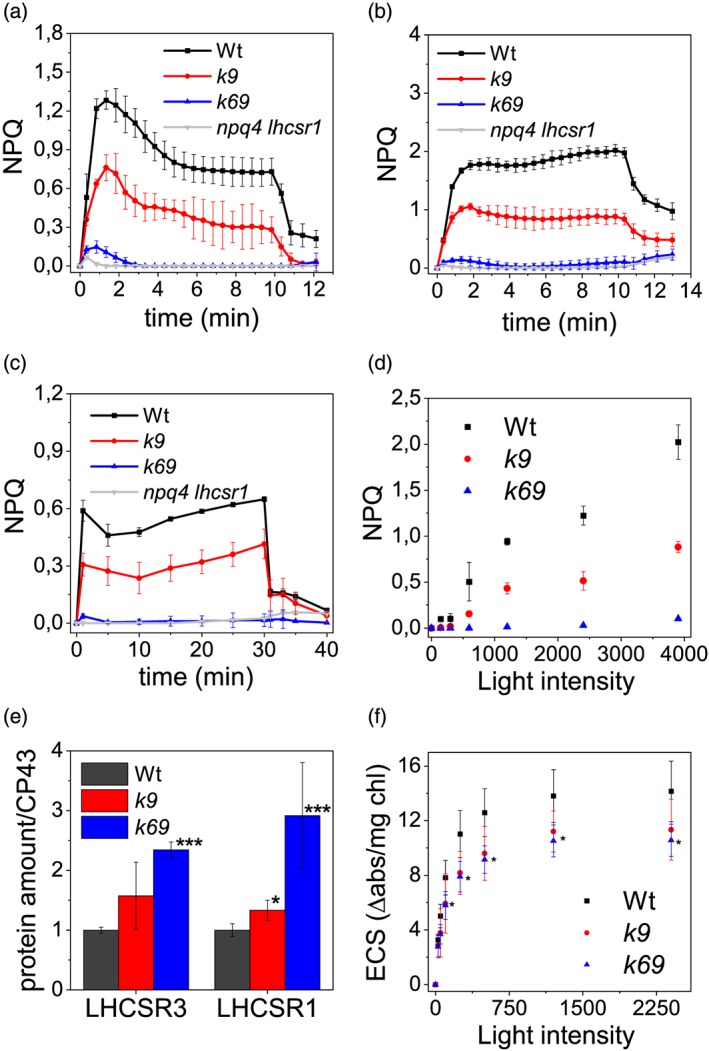
Nonphotochemical quenching (NPQ), LHCSR subunits accumulation, and proton transport. Measurement of NPQ kinetic on wild‐type (Wt, black), *k9* (red), *k69* (blue), and *npq4 lhcsr1* (grey) cells using actinic lights of (a) 1,200‐μmol photons m^−2^ s^−1^ and (b) 3,900‐μmol photons m^−2^ s^−1^ for 10 min of illumination. (c) NPQ kinetic using actinic lights of 1,200‐μmol photons m^−2^ s^−1^ for 30 min. (d) NPQ value after 10 min of illumination at different actinic light intesities. (e) LHCSR and CP43 amounts were quantified by immunotitration. LHCSR/CP43 ratios were normalized to the Wt value. (f) Estimation of total proton motive force upon exposure to different light intensities, measured as electrochromic shift (ECS) of carotenoid absorption spectrum. Data are expressed as mean ± *SD* (*n* = 4). ^*^Values that are significantly different (Student's *t* test, *P* < 0.05) from Wt. ^**^Data that are significantly different between *k6* and *k69*

Figure 5 shows the NPQ of the three genotypes: *k9* mutant showed a halved NPQ value compared with Wt, whereas surprisingly, *k69* double mutant showed no NPQ like the *npq4 lhsr1* mutant, the only difference being that in the first minute, *k69* presented a transient NPQ rise (Figures 5a and S9). Even when NPQ induction was tested with longer illumination times or at higher actinic light (Figure 5b,c), the double mutant was still unable to activate NPQ, whereas the *k9* mutant was characterized by an intermediate phenotype (Figure 5d).

The activation of NPQ in *C. reinhardtii* depends on lumen acidification and on accumulation of LHCSR proteins: We verified that both of these mechanisms were still active in mutants. LHCSR1 and LHCSR3 were present in mutants, and their abundance, with respect to PSII core, was even higher than in Wt (Figures 5 and S10). The generation of an electrochemical proton gradient across thylakoid membranes was assessed using ECS‐induced absorbance changes at 520 nm (Figure 5f; Bailleul, Cardol, Breyton, & Finazzi, 2010; Lucker & Kramer, 2013). The ECS, measured at the different light intensities, was similar between Wt and *k9* and only slightly reduced in the double‐mutant *k69*. Because LHCSR proteins were present and lumen acidification is not dramatically reduced, changes in NPQ activity are expected to reflect mainly an altered efficiency of quenching reactions in the absence of CP29 and CP26.

**Figure 6:**
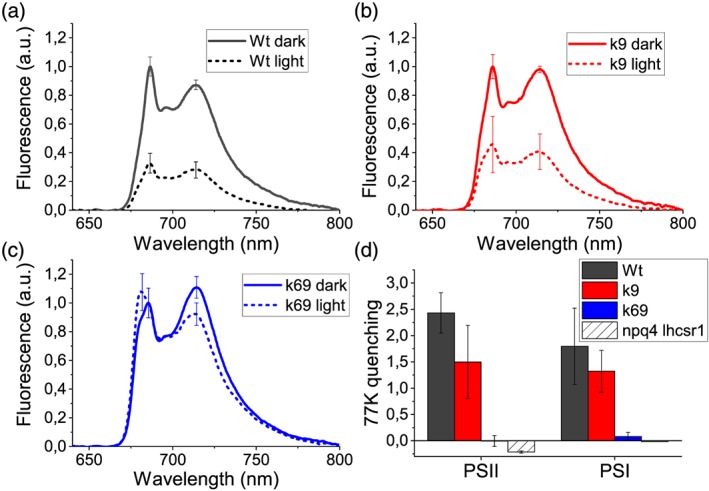
Measurement of quenching 77‐K fluorescence emission spectroscopy. Low‐temperature fluorescence emission spectra of (a) wild type, (b) *k9*, and (c) *k69* dark adapted (dark) or illuminated for 10 min at 1,200‐μmol photons m^−2^ s^−1^ (light) normalized to green fluorescent protein signal (see Section 2 for details). Cells were excited at 475 nm. (d) Calculated PSII and PSI quenching from the Gaussians used for fitting the two peaks according to the formula (*A*_Dark_ − *A*_light_)/*A*_light_, where *A*_Dark_ and *A*_light_ are, respectively, the amplitude of fuorescence emission in the dark and after illumination. Data are expressed as mean ± *SD* (*n* = 4)

To confirm the absence of quenching in the monomeric antenna double mutant, NPQ induction was assessed with an alternative method, more specifically by looking at low‐temperature (77 K) fluorescence prior to or at the end of actinic illumination using the green fluorescent protein as an internal standard for fluorescence emission spectra normalization (Girolomoni et al., 2019; Figure 6). A 77‐K fluorescence also allowed the determination of PSI quenching, not detectable at room temperature with PAM measurement. In Wt and *k9* mutant, upon illumination, the amplitude of the fluorescence spectra decreased involving both PSII and PSI components. In *k69*, double‐mutant PSII fluorescence emission did not decrease, whereas only a minor reduction was detected on PSI peak at ~710 nm. Deconvolution of the 77‐K fluorescence emission spectra with Gaussian functions allowed to distinctly evaluate the quenching on PSII and PSI (Figures 6d and S11). This analysis confirmed the quenching of both PSII and PSI in Wt and *k9*, whereas there was no significant quenching in the *k69* double mutant, neither for PSII nor for PSI. These data point out that in *C. reinhardtii*, even if LHCSR subunits are present, NPQ of PSI and PSII cannot be activated in the absence of both CP26 and CP29.

## 4 DISCUSSION

*C. reinhardtii cp29* and *cp26* genes were targeted by CRISPR‐Cas9 technology to obtain mutants deprived of one or both PSII monomeric antenna subunits. The first surprising result in the characterization of these mutants was the phenotype of *k6* lines, lacking not only CP26 but also CP29 subunit (Figure 1). Eight distinct *k6* lines were analysed, and all confirmed that the deletion of CP26 also caused the loss of CP29, whereas the transcription of *cp29* gene was not affected (Figure 1). These results imply that the loss of CP29 in *k6* lines was related to translational or post‐translational events: Likely, the removal of CP26 decreased CP29 stability in the PSII supercomplexes causing CP29 degradation. The inner layer of PSII antenna is composed of CP29, CP26, and S‐LHCII, forming with the core the C2S2 particle (Caffarri, Kouril, Kereiche, Boekema, & Croce, 2009): The absence of CP26 could weaken the binding of S‐LHCII to the core, and the absence of this trimer could thereby destabilize the CP29 connection to the core. An analogue effect was noted for CP24 in higher plants, where CP29 knock‐out plants lacked also CP24: CP24 and CP29 co‐operate in binding M‐LHCII (moderately bound) to the core, forming the C_2_S_2_M_2_ complex (de Bianchi et al., 2011). The fact that CP26 was still present in *k9* mutant could indicate that CP26 could, by itself, maintain a stable connection between S‐LHCII and the core even in the absence of CP29. Alternatively, because CP26 is in an external position in the PSII supercomplex (Drop, Webber‐Birungi, et al., 2014), it could be stabilized by the interaction with antennas of interacting neighbour PSII complexes.

The increase in F_0_ and the blue shift observed in the first peak of 77‐K fluorescence emission spectra indicate that, in the absence of CP26 and CP29, LHCII trimers are badly connected to PSII core and a larger fraction of absorbed energy is lost as fluorescence in the mutant instead of being efficiently harvested for photochemistry as already observed for monomeric antenna mutants in higher plants (Dall'Osto, Unlu, Cazzaniga, & Van Amerongen, 2014; de Bianchi et al., 2011; Townsend et al., 2018). The defective connection of the PSII antenna in the mutant was also highlighted by the separation of photosynthetic complexes on a sucrose gradient, where the lower band, corresponding to PSII supercomplex, was essentially absent in the *k9* and *k69* mutants (Figure 2).

Both *k9* and *k69* mutants present an increased LHCII content and thus an increased functional antenna size of PSII: In the absence of monomeric subunits located at the interface between LHCII‐M and LHCII‐S, the excitation energy transfer from LHCII to PSII core is likely to be reduced, stimulating the partial compensatory increase of LHCII antenna amount. This is similar to what has been observed in plants in limiting light conditions, where LHCII accumulation is increased (Kouril, Wientjes, Bultema, Croce, & Boekema, 2013). However, this strategy was only partially successful in the absence of CP29 and CP26: *k69* mutant showed an approximately fivefold increase in LHCII/PSII stoichiometry, but the functional antenna size was only increased by approximately twofold with respect to Wt. Moreover, it is worth to note that the functional antenna size of *k9* and *k69* might be overestimated as a consequence of the increased F_0_ observed in these strains (Malkin et al., 1981). These findings thus demonstrate that in the absence of CP26 and CP29, a significant fraction of trimers was not energetically coupled with PSII. Monomeric antenna proteins have thus a key role in *C. reinhardtii* in controlling excitation energy transfer from LHCII to the reaction centre, as previously reported in the case of higher plants (Townsend et al., 2018; van Amerongen & Croce, 2013). Values of PSII operating efficiency, relative ETR, ECS, and oxygen evolution were lower at all light intensities tested: The absence of CP29 or both CP26 and CP29 caused a partial reduction of photosynthetic efficiency, which could not be restored by the increased content of LHCII (Figure 3).

Previous work showed that CP29 is involved also in state transitions (Tokutsu et al., 2009) and more specifically in the docking of LHCII to PSI (Drop, Yadav K N, et al., 2014; Kargul et al., 2005). The results herein reported demonstrate that in *C. reinhardtii*, both CP26 and CP29 are involved in state transitions (Tokutsu et al., 2009), with a no‐state transitions phenotype for the *k69* mutant. Indeed, it has been shown that both subunits in State 2 condition are phosphorylated and likely bind to PSI with LHCII trimers upon State 2 induction (Takahashi, Iwai, Takahashi, & Minagawa, 2006).

The results herein presented confirm the important role of monomeric antenna subunits in light harvesting and in preserving PSII photosynthetic efficiency. Similar evidence has already been reported in *A. thaliana* (Dall'Osto et al., 2017; Townsend et al., 2018), even if the effects were more extreme in *C. reinhardtii* with respect to higher plant. The stronger difference observed when comparing *C. reinhardtii* and *A. thaliana* mutants depleted of monomeric CP26 and CP29 subunits is the NPQ phenotype. In an *A. thaliana* mutant without monomeric antennae, NPQ activation was slower in dark‐to‐light transition, but after a few minutes of illumination, the quenching was identical to Wt (Dall'Osto et al., 2017; Townsend et al., 2018). In the case of *C. reinhardtii*, in the absence of CP29, an almost halved NPQ phenotype was detected, whereas the double‐mutant *k69* presented a no‐NPQ phenotype even after 30 min of illumination at high light intensity (Figure 5). In *C. reinhardtii*, NPQ is thus totally dependent on the presence of PSII monomeric antenna subunits. The NPQ phenotype observed in *k9* and *k69* strains is not related to a different accumulation of LHCSR subunits, the NPQ triggers, because these subunits were rather increased in the absence of CP26 and CP29. Moreover, the absence of NPQ could not be due to a different LHCSR/antenna stoichiometry because in high light adapted cells, the difference in Chl a/b ratio between Wt and *k69* was only ~10%. *k69* mutants exhibited also an impairment in state transitions induction and a slight reduction of the amount of zeaxanthin accumulated after 2 weeks in high light (Table S2). However, these differences cannot be responsible for the no‐NPQ phenotype observed in the absence of CP26 and CP29 subunits: Both *stt7* and *npq1* mutants, impaired in state transitions and zeaxanthin accumulation, respectively, were reported with a similar NPQ compared with Wt (Allorent et al., 2013; Girolomoni et al., 2019; Niyogi, Björkman, & Grossman, 1997). *k69* mutant has reduced electron transport rates, oxygen evolution, and proton gradient generation, but these alterations could eventually explain a slower kinetic of NPQ activation but not its complete absence after prolonged illumination (Townsend et al., 2018). Besides, the highest actinic light used to induce NPQ was at least three times higher than the intensity needed to saturate photosynthesis, according to the oxygen evolution curves reported in Figure 3. The complete absence of NPQ in the absence of CP26 and CP29 is thus related to a specific role of monomeric subunits in the mechanism of NPQ and/or their interaction with LHCSR subunits. The NPQ phenotype in *k9* and *k69* mutants suggests that LHCSR subunits need to interact with CP26 and CP29 to act as quenchers upon NPQ induction, even if it could be excluded that CP29 and/or CP26 might be activated as quenchers themselves by LHCSR when it is protonated. It has been suggested that CP26 and an LHCII trimers are the docking sites of LHCSR3 on PSII. Our results demonstrate that in the absence of CP29, a strong reduction of NPQ is evident, suggesting a direct interaction between LHCSR subunit and CP29 or an excitation energy transfer from CP29 to LHCSR subunits through an LHCII trimer. In the absence of both CP26 and CP29, the NPQ induction is impaired, demonstrating that the interaction between LHCSR3 and LHCII trimers is not sufficient to induce NPQ. Because we could not obtain a single mutant only without CP26, it is not possible to confirm if the NPQ of the double mutant was due to the absence of both subunits or to the absence of CP26 exclusively. Additional work is required to investigate the specific role of CP26. Considering the destabilization of PSII‐LHCII supercomplexes in the absence of CP26 and CP29, an alternative possibility explaining the NPQ phenotype of *k9* and *k69* strains is an indirect role of CP26 and CP29, which the absence might destabilize some other PSII subunits important for LHCSR1/LHCSR3 binding as quenching activity, as reported in the case of PSBR (Xue et al., 2015).

Recent reports have pointed out that LHCSR also acts as quencher of PSI through the associated LHCII antennas (Girolomoni et al., 2019; Kosuge et al., 2018): This quenching component was also absent in *k69*. Considering that both CP26 and CP29 are involved in LHCII detachment from PSII and its migration towards PSI, it is possible that LHCSR‐dependent quenching of LHCII, when detached from PSII and/or connected to PSI, may be also related to LHCSR interaction with monomeric antennas. Recently, a megacomplex composed of PSII, PSI, PsbS, and LHCSR was reported in the case of *Physcomitrella patens* (Furukawa et al., 2019): The formation of such megacomplexes in *C. reinhardtii* and their possible role in PSI quenching needs to be investigated by additional work.

The specific role of CP26 and CP29 in state transitions and NPQ induction herein described reveals a strong difference in the function of monomeric antenna proteins among *A. thaliana* and *C. reinhardtii*. In plant, monomeric antenna are not essential for NPQ, and their absence does not abolish state transitions (Dall'Osto et al., 2017; Townsend et al., 2018), whereas in the latter, both adaptive mechanisms require the presence of CP26 and/or CP29.

In conclusion, the data herein reported expand the understanding of the divergence between green algae and higher plants of light harvesting and photoprotection mechanisms. This could be the basis for further research work to tune and increase microalgae biomass productivity to be used as food or energy source. Any effort towards microalgae domestication should likely avoid altering monomeric antenna subunits for their essential role in light conversion efficiency and photoprotective mechanisms.

## CONFLICT OF INTEREST

Authors declare no conflict of interest.

## Supporting information


**Figure S1.** **Chromatograms of ΔCP29 mutants at the *cp29* locus and CP29 protein sequences predicted to be translated.** Red color indicates disrupted region by indel mutation in the CP29 protein. WT CP29 protein is composed by 280 residues
**Figure S2. Absence of CP29 protein in *k6* lines.** Total protein extract for 8 different knock‐out lines for CP26 (*k6.1‐k6.8*) were loaded on SDS‐page gel and checked for the presence of CP29 by immunoblot with specific antibody. Immunoblot against CP43 was added as control of the loading, immunoblot against CP26 to confirm that *k6* strains were knock‐out lines for CP26. Total protein extract from wild‐type (Wt) and a *k9* lines were added on the external lanes as control.
**Figure S3. qRT‐PCR on cp26,cp29 and rack 1 gene.** a, Sequence of primers used for amplification of *cp26* and *cp29* CDS (Fig. 1b) and for cp26, CP29 and rack1 qRT‐PCR. b, Scheme of primers pairing on *cp26* and *cp29* genes. sgRNA target are reported in blue color. c, qRT on *rack1* gene used as loading control for qRT‐PCR on *cp26* and *cp29* genes reported in Fig.1
**Figure S4. Polypeptide composition of thylakoid membranes**. Image of two of the Western blot used for Immunotitration of thylakoid proteins in Fig. 2b. Specific antibodies against PSAA, CP43, LHCII and LHCA were used on cellulose on lanes loaded with 2, 1, 0.5 and 0.25 μg of Chls. On each gel wild‐type (Wt) thylakoids were loaded in order to normalize the data.
**Figure S5. Densitometric analysis of sucrose gradients**. Sucrose gradient loaded with solubilized thylakoids were analysed by densitometric analysis with GelPro extracting on green channel. Densitometric results are reported as optical density (OD) normalized to the total green of each gradient.
**Figure S6. Functional PSII antenna size.** Variable Chl fluorescence was induced with a weak red light of 11 μmol photons m‐2 s‐1, on dark‐adapted cells (about 2 · 106 cells/ml) in HS medium supplemented with 50 μM DCMU. The trace for wild‐type (Wt, black), k9 (grey) and k69 (light grey) are the average of 40 curve for each genotype from four different experiments. The reciprocal of time corresponding to two‐thirds of the fluorescence rise (1/τ2/3) is as a measure of the PSII functional antenna size and it is shown in the inset and in Table 1 normalized to the WT case, which was set to 100. Data are expressed as mean ± SD. Values that are significantly different (Student's t‐test, P < 0.05) from the wild‐type (WT) are marked with an asterisk (*). Date that are significantly different between k6 and k69 are marked with a circle (°).
**Figure S7. Western blot analysis of STT7 enzyme in Wt and mutant strains.** Western blot were performed on STT7 kinase and CP43 used as loading control.
**Figure S8. Quantification of minimal and maximum Chl fluorescence.** The same amount of dark‐adapted wild‐type (Wt), k9 and k69 cells (2 · 106 cells/ml) was excited with same PAM light setting and minimal (F0) and maximal (Fm) were recorded. After the measure Chl were extracted and quantified from all the sample. F0 and Fm were normalized to cells (**a,b** for F0 and Fm respectively) and Chl content (**c,d** for F0 and Fm respectively). Data are expressed as mean ± SD. Values that are significantly different (Student's t‐test, P < 0.05) from the Wt are marked with an asterisk (*).
**Figure S9. NPQ chlorophyll fluorescence.** Example of fluorescence traces from wild‐type (Wt black), k9 (grey) and k69 (light grey) obtained from NPQ measure using actinic light of 1200 μmol photons m‐2 s‐1. Traces are vertically shifted to the same value of Fm.
**Figure S10. LHCSR quantification**. Image of two of the Western blot used for immunotitration of thylakoid proteins in Fig. 5a. Specific antibodies against LHCSR3 and LHCSR1 were used on lanes loaded with 2, 1, 0,5 and 0,25 μg of Chl. On each gel wild‐type (Wt) thylakoids were loaded in order to normalize the data. *npq4 lhcsr1* thylakoid (1 μg of Chl) was loaded as negative control.
**Figure S11. 77K fluorescence of WT and mutant strains.** Low temperature fluorescence emission spectra of dark‐adapted (dark) or light treated (light) wild‐type (a,b), *k9* (c,d), *k69* (e,f) and *npq4 lhcsr1*(g,h) cells, shown if figure 7, were reconstructed by spectral deconvolution with Gaussians. Cumulative fit results are reported in red.
**Table S1. Photosynthesis and respiration rates**
**Table S2. Pigment content of cell acclimated to 500 μmol photons m‐2 s‐1.**
**Table S3. Fv/Fm of cell acclimated to 500 μmol photons m‐2 s‐1.**

## References

[pce13680-bib-0001] Ahn, T. K. , Avenson, T. J. , Ballottari, M. , Cheng, Y. C. , Niyogi, K. K. , Bassi, R. , & Fleming, G. R. (2008). Architecture of a charge‐transfer state regulating light harvesting in a plant antenna protein. Science, 320(5877), 794–797. 10.1126/science.1154800 18467588

[pce13680-bib-0002] Alboresi, A. , Gerotto, C. , Giacometti, G. M. , Bassi, R. , & Morosinotto, T. (2010). *Physcomitrella patens* mutants affected on heat dissipation clarify the evolution of photoprotection mechanisms upon land colonization. Proceedings of the National Academy of Sciences of the United States of America, 107(24), 11128–11133. 20505121 10.1073/pnas.1002873107 PMC2890724

[pce13680-bib-0003] Allen, J. F. (1992). Protein phosphorylation in regulation of photosynthesis. Biochimica et Biophysica Acta, 1098, 275–335. 10.1016/s0005-2728(09)91014-3 1310622

[pce13680-bib-0004] Allorent, G. , Lefebvre‐Legendre, L. , Chappuis, R. , Kuntz, M. , Truong, T. B. , Niyogi, K. K. , … Goldschmidt‐Clermont, M. (2016). UV‐B photoreceptor‐mediated protection of the photosynthetic machinery in *Chlamydomonas reinhardtii* . Proceedings of the National Academy of Sciences of the United States of America, 113(51), 14864–14869. 10.1073/pnas.1607695114 27930292 PMC5187749

[pce13680-bib-0005] Allorent, G. , Tokutsu, R. , Roach, T. , Peers, G. , Cardol, P. , Girard‐Bascou, J. , … Finazzi, G. (2013). A dual strategy to cope with high light in *Chlamydomonas reinhardtii* . Plant Cell, 25(2), 545–557. 10.1105/tpc.112.108274 23424243 PMC3608777

[pce13680-bib-0006] Baek, K. , Kim, D. H. , Jeong, J. , Sim, S. J. , Melis, A. , Kim, J. S. , … Bae, S. (2016). DNA‐free two‐gene knockout in *Chlamydomonas reinhardtii* via CRISPR‐Cas9 ribonucleoproteins. Scientific Reports, 6(30620), 1–7. 10.1038/srep30620 27466170 PMC4964356

[pce13680-bib-0007] Bailleul, B. , Cardol, P. , Breyton, C. , & Finazzi, G. (2010). Electrochromism: A useful probe to study algal photosynthesis. Photosynthesis Research, 106(1‐2), 179–189. 20632109 10.1007/s11120-010-9579-z

[pce13680-bib-0008] Baker, N. R. (2008). Chlorophyll fluorescence: A probe of photosynthesis in vivo. Annual Review of Plant Biology, 59, 89–113. 10.1146/annurev.arplant.59.032607.092759 18444897

[pce13680-bib-0009] Ballottari, M. , Dall'Osto, L. , Morosinotto, T. , & Bassi, R. (2007). Contrasting behavior of higher plant photosystem I and II antenna systems during acclimation. Journal of Biological Chemistry, 282(12), 8947–8958. 17229724 10.1074/jbc.M606417200

[pce13680-bib-0010] Ballottari, M. , Girardon, J. , Dall'Osto, L. , & Bassi, R. (2012). Evolution and functional properties of photosystem II light harvesting complexes in eukaryotes. Biochimica et Biophysica Acta‐Bioenergetics, 1817(1), 143–157. 10.1016/j.bbabio.2011.06.005 21704018

[pce13680-bib-0011] Bonente, G. , Passarini, F. , Cazzaniga, S. , Mancone, C. , Buia, M. C. , Tripodi, M. , … Caffarri, S. (2008). The occurrence of the *psbS* gene product in *Chlamydomonas reinhardtii* and in other photosynthetic organisms and its correlation with energy quenching. Photochemistry and Photobiology, 84(6), 1359–1370. 10.1111/j.1751-1097.2008.00456.x 19067957

[pce13680-bib-0012] Butler, W. L. (1973). Primary photochemistry of photosystem II in photosynthesis. Accounts of Chemical Research, 6, 177–183.

[pce13680-bib-0013] Caffarri, S. , Kouril, R. , Kereiche, S. , Boekema, E. J. , & Croce, R. (2009). Functional architecture of higher plant photosystem II supercomplexes. The EMBO Journal, 28, 3052–3063. 19696744 10.1038/emboj.2009.232 PMC2760109

[pce13680-bib-0014] Correa‐Galvis, V. , Redekop, P. , Guan, K. , Griess, A. , Truong, T. B. , Wakao, S. , … Jahns, P. (2016). Photosystem II subunit PsbS is involved in the induction of LHCSR protein‐dependent energy dissipation in *Chlamydomonas reinhardtii* . The Journal of Biological Chemistry, 291(33), 17478–17487. 10.1074/jbc.M116.737312 27358399 PMC5016143

[pce13680-bib-0015] Croce, R. , Canino, G. , Ros, F. , & Bassi, R. (2002). Chromophore organization in the higher‐plant photosystem II antenna protein CP26. Biochemistry, 41(23), 7334–7343. 10.1021/bi0257437 12044165

[pce13680-bib-0016] Croce, R. , & van Amerongen, H. (2013). Light‐harvesting in photosystem I. Photosynthesis Research, 116, 153–166. 10.1007/s11120-013-9838-x 23645376 PMC3825136

[pce13680-bib-0017] Dall'Osto, L. , Cazzaniga, S. , Bressan, M. , Paleček, D. , Židek, K. , Niyogi, K. K. , … Bassi, R. (2017). Two mechanisms for dissipation of excess light in monomeric and trimeric light‐harvesting complexes. Nature Plants, 3(17033), 1–9. 10.1038/nplants.2017.33 28394312

[pce13680-bib-0018] Dall'Osto, L. , Unlu, C. , Cazzaniga, S. , & Van Amerongen, H. (2014). Disturbed excitation energy transfer in *Arabidopsis thaliana* mutants lacking minor antenna complexes of photosystem II. Biochimica et Biophysica Acta, 1837(12), 1981–1988. 25291424 10.1016/j.bbabio.2014.09.011

[pce13680-bib-0019] de Bianchi, S. , Betterle, N. , Kouril, R. , Cazzaniga, S. , Boekema, E. , Bassi, R. , & Dall'Osto, L. (2011). Arabidopsis mutants deleted in the light‐harvesting protein *Lhcb4* have a disrupted photosystem II macrostructure and are defective in photoprotection. Plant Cell, 23(7), 2659–2679. 10.1105/tpc.111.087320 21803939 PMC3226214

[pce13680-bib-0020] Depege, N. , Bellafiore, S. , & Rochaix, J. D. (2003). Role of chloroplast protein kinase *Stt7* in LHCII phosphorylation and state transition in Chlamydomonas. Science, 299(5612), 1572–1575. 10.1126/science.1081397 12624266

[pce13680-bib-0021] Drop, B. , Webber‐Birungi, M. , Yadav, S. K. , Filipowicz‐Szymanska, A. , Fusetti, F. , Boekema, E. J. , & Croce, R. (2014). Light‐harvesting complex II (LHCII) and its supramolecular organization in *Chlamydomonas reinhardtii* . Biochimica et Biophysica Acta, 1837(1), 63–72. 10.1016/j.bbabio.2013.07.012 23933017

[pce13680-bib-0022] Drop, B. , Yadav, K. N. S. , Boekema, E. J. , & Croce, R. (2014). Consequences of state transitions on the structural and functional organization of photosystem I in the green alga *Chlamydomonas reinhardtii* . The Plant Journal, 78(2), 181–191. 10.1111/tpj.12459 24506306

[pce13680-bib-0023] Furukawa, R. , Aso, M. , Fujita, T. , Akimoto, S. , Tanaka, R. , Tanaka, A. , … Takabayashi, A. (2019). Formation of a PSI–PSII megacomplex containing LHCSR and PsbS in the moss *Physcomitrella patens* . Journal of Plant Research, 132(6), 867–880. 10.1007/s10265-019-01138-2 31541373

[pce13680-bib-0024] Garnier, J. , Maroc, J. , & Guyon, D. (1986). Low‐temperature fluorescence emission spectra and chlorophyll–protein complexes in mutants of *Chlamydomonas reinhardtii*: Evidence for a new chlorophyll‐a‐protein complex related to photosystem I. Biochimica et Biophysica Acta, 851, 395–406.

[pce13680-bib-0025] Genty, B. , Briantais, J.‐M. , & Baker, N. R. (1989). The relationship between the quantum yield of photosynthetic electron transport and quenching of chlorophyll fluorescence. Biochimica et Biophysica Acta, 990, 87–92.

[pce13680-bib-0026] Girolomoni, L. , Cazzaniga, S. , Pinnola, A. , Perozeni, F. , Ballottari, M. , & Bassi, R. (2019). LHCSR3 is a nonphotochemical quencher of both photosystems in *Chlamydomonas reinhardtii* . Proceedings of the National Academy of Sciences of the United States of America, 116(10), 4212–4217. 10.1073/pnas.1809812116 30782831 PMC6410775

[pce13680-bib-0027] Harris, E. H. , & Harris (2008). Introduction to Chlamydomonas and its laboratory use (Vol. 1). San Diego: Academic press.

[pce13680-bib-0028] Hill, R. , & Scarisbrick, R. (1940). Production of oxygen by illuminated chloroplasts. Nature, 146, 61–62. 10.1038/146061a0

[pce13680-bib-0029] Jansson, S. (1999). A guide to the Lhc genes and their relatives in Arabidopsis. Trends in Plant Science, 4, 236–240. 10366881 10.1016/s1360-1385(99)01419-3

[pce13680-bib-0030] Kargul, J. , Turkina, M. V. , Nield, J. , Benson, S. , Vener, A. V. , & Barber, J. (2005). Light‐harvesting complex II protein CP29 binds to photosystem I of *Chlamydomonas reinhardtii* under State 2 conditions. The FEBS Journal, 272(18), 4797–4806. 10.1111/j.1742-4658.2005.04894.x 16156798

[pce13680-bib-0031] Kosuge, K. , Tokutsu, R. , Kim, E. , Akimoto, S. , Yokono, M. , Ueno, Y. , & Minagawa, J. (2018). LHCSR1‐dependent fluorescence quenching is mediated by excitation energy transfer from LHCII to photosystem I in. Proceedings of the National Academy of Sciences of the United States of America, 115, 3722–3727. 10.1073/pnas.1720574115 29555769 PMC5889656

[pce13680-bib-0032] Kouril, R. , Wientjes, E. , Bultema, J. B. , Croce, R. , & Boekema, E. J. (2013). High‐light vs. low‐light: Effect of light acclimation on photosystem II composition and organization in *Arabidopsis thaliana* . Biochimica et Biophysica Acta‐Bioenergetics, 1827(3), 411–419. 10.1016/j.bbabio.2012.12.003 23274453

[pce13680-bib-0033] Kuhlgert, S. , Austic, G. , Zegarac, R. , Osei‐Bonsu, I. , Hoh, D. , Chilvers, M. I. , … Kramer, D. M. (2016). MultispeQ Beta: A tool for large‐scale plant phenotyping connected to the open PhotosynQ network. Royal Society Open Science, 3(10), 1–17. 10.1098/rsos.160592 PMC5099005 27853580

[pce13680-bib-0034] Lagarde, D. , Beuf, L. , & Vermaas, W. (2000). Increased production of zeaxanthin and other pigments by application of genetic engineering techniques to *Synechocystis* sp. strain PCC 6803. Applied and Environmental Microbiology, 66(1), 64–72. 10.1128/aem.66.1.64-72.2000 10618204 PMC91786

[pce13680-bib-0035] Li, X. P. , Gilmore, A. M. , Caffarri, S. , Bassi, R. , Golan, T. , Kramer, D. , & Niyogi, K. K. (2004). Regulation of photosynthetic light harvesting involves intrathylakoid lumen pH sensing by the PsbS protein. The Journal of Biological Chemistry, 279(22), 22866–22874. 15033974 10.1074/jbc.M402461200

[pce13680-bib-0036] Liu, Z. , Yan, H. , Wang, K. , Kuang, T. , Zhang, J. , Gui, L. , … Chang, W. (2004). Crystal structure of spinach major light‐harvesting complex at 2.72 A resolution. Nature, 428(6980), 287–292. 10.1038/nature02373 15029188

[pce13680-bib-0037] Lucker, B. , & Kramer, D. M. (2013). Regulation of cyclic electron flow in *Chlamydomonas reinhardtii* under fluctuating carbon availability. Photosynthesis Research, 117(1‐3), 449–459. 10.1007/s11120-013-9932-0 24113925

[pce13680-bib-0038] Malkin, S. , Armond, P. A. , Mooney, H. A. , & Fork, D. C. (1981). Photosystem II photosynthetic unit sizes from fluorescence induction in leaves: Correlation to photosynthetic capacity. Plant Physiology, 67(3), 570–579. 10.1104/pp.67.3.570 16661716 PMC425727

[pce13680-bib-0039] Murchie, E. H. , & Niyogi, K. K. (2011). Manipulation of photoprotection to improve plant photosynthesis. Plant Physiology, 155(1), 86–92. 10.1104/pp.110.168831 21084435 PMC3075776

[pce13680-bib-0040] Niyogi, K. K. , Björkman, O. , & Grossman, A. R. (1997). Chlamydomonas xanthophyll cycle mutants identified by video imaging of chlorophyll fluorescence quenching. Plant Cell, 9, 1369–1380. 12237386 10.1105/tpc.9.8.1369 PMC157004

[pce13680-bib-0041] Peers, G. , Truong, T. B. , Ostendorf, E. , Busch, A. , Elrad, D. , Grossman, A. R. , … Niyogi, K. K. (2009). An ancient light‐harvesting protein is critical for the regulation of algal photosynthesis. Nature, 462, 518–521. 10.1038/nature08587 19940928

[pce13680-bib-0042] Ruban, A. V. , Berera, R. , Ilioaia, C. , van Stokkum, I. H. , Kennis, J. T. , Pascal, A. A. , … van Grondelle, R. (2007). Identification of a mechanism of photoprotective energy dissipation in higher plants. Nature, 450(7169), 575–578. 10.1038/nature06262 18033302

[pce13680-bib-0043] Schägger, H. , & von Jagow, G. (1987). Tricine‐sodium dodecyl sulfate‐polyacrylamide gel electrophoresis for the separation of proteins in the range from 1 to 100 kDa. Analytical Biochemistry, 166, 368–379. 10.1016/0003-2697(87)90587-2 2449095

[pce13680-bib-0044] Scholz, M. , Gäbelein, P. , Xue, H. , Mosebach, L. , Bergner, S. V. , & Hippler, M. (2019). Light‐dependent N‐terminal phosphorylation of LHCSR3 and LHCB4 are interlinked in *Chlamydomonas reinhardtii* . The Plant Journal, 99(5), 877–894. 10.1111/tpj.14368 31033075 PMC6851877

[pce13680-bib-0045] Semchonok, D. A. , Sathish Yadav, K. N. , Xu, P. , Drop, B. , Croce, R. , & Boekema, E. J. (2017). Interaction between the photoprotective protein LHCSR3 and C2S2 photosystem II supercomplex in *Chlamydomonas reinhardtii* . Biochimica et Biophysica Acta, 1858(5), 379–385. 10.1016/j.bbabio.2017.02.015 28257778

[pce13680-bib-0046] Shin, Y. S. , Jeong, J. , Nguyen, T. H. T. , Kim, J. Y. H. , Jin, E. , & Sim, S. J. (2019). Targeted knockout of phospholipase A2 to increase lipid productivity in *Chlamydomonas reinhardtii* for biodiesel production. Bioresource Technology, 271, 368–374. 10.1016/j.biortech.2018.09.121 30293032

[pce13680-bib-0047] Suga, M. , Akita, F. , Hirata, K. , Ueno, G. , Murakami, H. , Nakajima, Y. , … Shen, J. R. (2015). Native structure of photosystem II at 1.95 A resolution viewed by femtosecond X‐ray pulses. Nature, 517(7532), 99–103. 10.1038/nature13991 25470056

[pce13680-bib-0048] Takahashi, H. , Iwai, M. , Takahashi, Y. , & Minagawa, J. (2006). Identification of the mobile light‐harvesting complex II polypeptides for state transitions in *Chlamydomonas reinhardtii* . Proceedings of the National Academy of Sciences of the United States of America, 103(2), 477–482. 16407170 10.1073/pnas.0509952103 PMC1326185

[pce13680-bib-0049] Tanksley, S. D. , Ganal, M. W. , Prince, J. P. , de Vicente, M. C. , Bonierbale, M. W. , Broun, P. , … Martin, G. B. (1992). High density molecular linkage maps of the tomato and potato genomes. Genetics, 132(4), 1141–1160. 1360934 10.1093/genetics/132.4.1141 PMC1205235

[pce13680-bib-0050] Tibiletti, T. , Auroy, P. , Peltier, G. , & Caffarri, S. (2016). *Chlamydomonas reinhardtii* PsbS protein is functional and accumulates rapidly and transiently under high light. Plant Physiology, 171(4), 2717–2730. 10.1104/pp.16.00572 27329221 PMC4972282

[pce13680-bib-0051] Tokutsu, R. , Iwai, M. , & Minagawa, J. (2009). CP29, a monomeric light‐harvesting complex II protein, is essential for state transitions in *Chlamydomonas reinhardtii* . The Journal of Biological Chemistry, 284(12), 7777–7782. 10.1074/jbc.M809360200 19144643 PMC2658071

[pce13680-bib-0052] Tokutsu, R. , Kato, N. , Bui, K. H. , Ishikawa, T. , & Minagawa, J. (2012). Revisiting the supramolecular organization of photosystem II in *Chlamydomonas reinhardtii* . The Journal of Biological Chemistry, 287(37), 31574–31581. 10.1074/jbc.M111.331991 22801422 PMC3438989

[pce13680-bib-0053] Townsend, A. J. , Saccon, F. , Giovagnetti, V. , Wilson, S. , Ungerer, P. , & Ruban, A. V. (2018). The causes of altered chlorophyll fluorescence quenching induction in the Arabidopsis mutant lacking all minor antenna complexes. Biochimica et Biophysica Acta ‐ Bioenergetics, 1859(9), 666–675. 10.1016/j.bbabio.2018.03.005 29548769

[pce13680-bib-0054] van Amerongen, H. , & Croce, R. (2013). Light harvesting in photosystem II. Photosynthesis Research, 116(2‐3), 251–263. 10.1007/s11120-013-9824-3 23595278 PMC3824292

[pce13680-bib-0055] Van Kooten, O. , & Snel, J. F. H. (1990). The use of chlorophyll fluorescence nomenclature in plant stress physiology. Photosynthesis Research, 25, 147–150. 24420345 10.1007/BF00033156

[pce13680-bib-0056] Vass, I. , Styring, S. , Hundal, T. , Koivuniemi, A. , Aro, E.‐M. , & Andersson, B. (1992). Reversible and irreversible intermediates during photoinhibition of photosystem II: Stable reduced QA species promote chlorophyll triplet formation. Proceedings of the National Academy of Sciences of the United States of America, 89, 1408–1412. 10.1073/pnas.89.4.1408 11607279 PMC48460

[pce13680-bib-0057] Xue, H. , Tokutsu, R. , Bergner, S. V. , Scholz, M. , Minagawa, J. , & Hippler, M. (2015). PHOTOSYSTEM II SUBUNIT R is required for efficient binding of LIGHT‐HARVESTING COMPLEX STRESS‐RELATED PROTEIN3 to photosystem II‐light‐harvesting supercomplexes in *Chlamydomonas reinhardtii* . Plant Physiology, 167(4), 1566–1578. 10.1104/pp.15.00094 25699588 PMC4378180

