# Supplementary material for: Photosystem II antenna complexes CP26 and CP29 are essential for nonphotochemical quenching in Chlamydomonas reinhardtii
Source: Plant Cell Environ. 2019 Dec 8;43(2):496–509. doi: 10.1111/pce.13680 (PMC7004014; doi:10.1111/pce.13680)
Supplement: Supplementary file 1 — Figure S1. Chromatograms of ΔCP29 mutants at the cp29 locus and CP29 protein sequences predicted to be translated. Red color indicates disrupted region by indel mutation in the CP29 protein. WT CP29 protein is composed by 280 residues Figure S2. Absence of CP29 protein in k6 lines. Total protein extract for 8 different knock‐out lines for CP26 (k6.1‐k6.8) were loaded on SDS‐page gel and checked for the presence of CP29 by immunoblot with specific antibody. Immunoblot against CP43 was added as control of the loading, immunoblot against CP26 to confirm that k6 strains were knock‐out lines for CP26. Total protein extract from wild‐type (Wt) and a k9 lines were added on the external lanes as control. Figure S3. qRT‐PCR on cp26,cp29 and rack 1 gene. a, Sequence of primers used for amplification of cp26 and cp29 CDS (Fig. 1b) and for cp26, CP29 and rack1 qRT‐PCR. b, Scheme of primers pairing on cp26 and cp29 genes. sgRNA target are reported in blue color. c, qRT on rack1 gene used as loading control for qRT‐PCR on cp26 and cp29 genes reported in Fig.1 Figure S4. Polypeptide composition of thylakoid membranes. Image of two of the Western blot used for Immunotitration of thylakoid proteins in Fig. 2b. Specific antibodies against PSAA, CP43, LHCII and LHCA were used on cellulose on lanes loaded with 2, 1, 0.5 and 0.25 μg of Chls. On each gel wild‐type (Wt) thylakoids were loaded in order to normalize the data. Figure S5. Densitometric analysis of sucrose gradients. Sucrose gradient loaded with solubilized thylakoids were analysed by densitometric analysis with GelPro extracting on green channel. Densitometric results are reported as optical density (OD) normalized to the total green of each gradient. Figure S6. Functional PSII antenna size. Variable Chl fluorescence was induced with a weak red light of 11 μmol photons m‐2 s‐1, on dark‐adapted cells (about 2 · 106 cells/ml) in HS medium supplemented with 50 μM DCMU. The trace for wild‐type (Wt, black), k9 (grey) and k69 (ligh [file PCE-43-496-s001.pdf]

Supporting Information

Photosystem II antenna complexes CP26 and CP29 are essential for non-photochemical quenching in *Chlamydomonas reinhardtii*

Stefano Cazzaniga[1#], Minjae Kim[2#], Francesco Bellamoli[1#], Jooyoen Jeong[2], Sangmuk Lee[2], Federico Perozeni[1], Andrea Pompa[3,4], EonSeon Jin[2*], Matteo Ballottari[1*]

[1] *Dipartimento di Biotecnologie, Università di Verona, Verona, Italy.*

[2] *Department of Life Science, Hanyang University, Seoul, South Korea*

[3] *Dipartimento di Scienze Biomolecolari, Università degli studi di Urbino*

[4] *Istituto di Bioscienze e Biorisorse, Consiglio Nazionale delle Ricerche, Perugia, Italy.*

[#] *These authors contributed equally to this work.*

*Address for correspondence: Matteo Ballottari, Dipartimento di Biotecnologie, Università di Verona, Strada le Grazie 15, 37134 Verona Italy; E-mail: matteo.ballottari@univr.it ; EonSeon Jin, Department of Life Science, Hanyang University, Seoul, South Korea esjin@hanyang.ac.kr

Figure S1. Chromatograms of ΔCP29 mutants at the *cp29* locus and CP29 protein sequences predicted to be translated. Red color indicates disrupted region by indel mutation in the CP29 protein. WT CP29 protein is composed by 280 residues

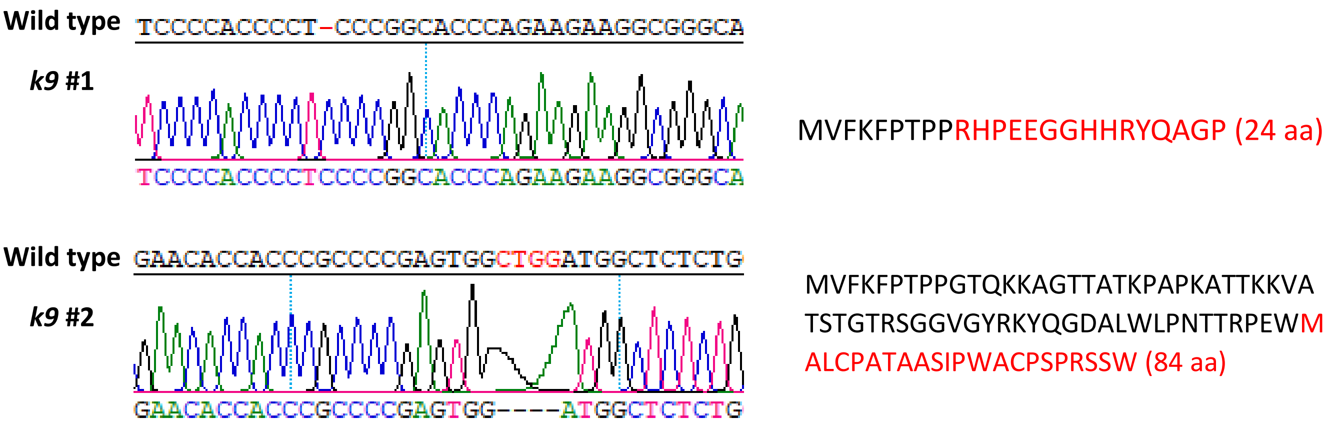

Figure S2. Absence of CP29 protein in *k6* lines. Total protein extract for 8 different knock-out lines for CP26 (*k6.1*-*k6.8*) were loaded on SDS-page gel and checked for the presence of CP29 by immunoblot with specific antibody. Immunoblot against CP43 was added as control of the loading, immunoblot against CP26 to confirm that *k6* strains were knock-out lines for CP26. Total protein extract from wild-type (Wt) and a *k9* lines were added on the external lanes as control.

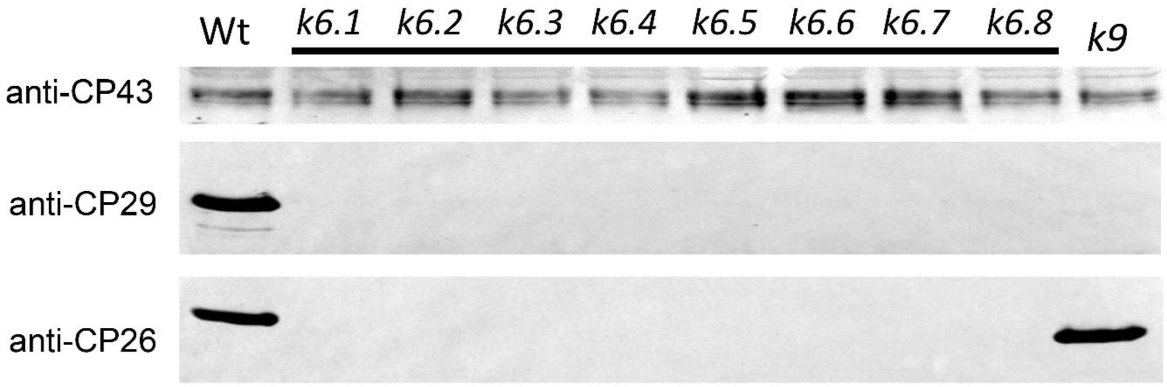

Figure S3. qRT-PCR on cp26,cp29 and rack 1 gene. a, Sequence of primers used for amplification of *cp26* and *cp29* CDS (Fig. 1b) and for cp26, CP29 and rack1 qRT-PCR. b, Scheme of primers pairing on *cp26* and *cp29* genes. sgRNA target are reported in blue color. c, qRT on *rack1* gene used as loading control for qRT-PCR on *cp26* and *cp29* genes reported in Fig.1

a

Primer sequences

cp26_CDS_F: ATGCAGATCCAGGCTCTGTT

cp26_CDS_R: TTACAGGGTGGGGGTGCGCT

cp29_CDS_F: ATGGTGTTCAAGTTCCCCAC

cp29_CDS_R: TTAGAGCCCCTTGCCGTTGT

qRT_cp26_246F: AGTTCGACTCGTCGGTCTTC

qRT_cp26_246R: GGACGGTCAGCAGGTTGTAG

qRT_cp29_250F: CCGGTGTGTCTTGGGTTGAG

qRT_cp29_250R: TGCTTGATCTCAGCGGTCTT

qRT_RACK1_F: GGCTGGGACAAGATGGTCAA

qRT_RACK1_R: GAGAAGCACAGGCAGTGGAT

b

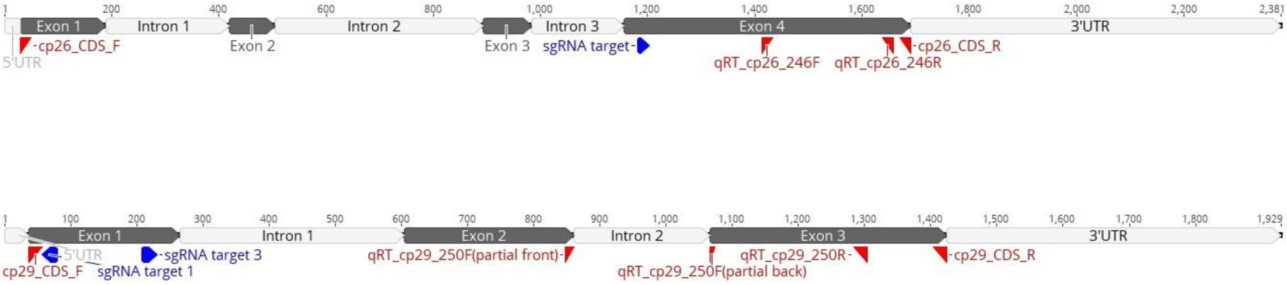

c

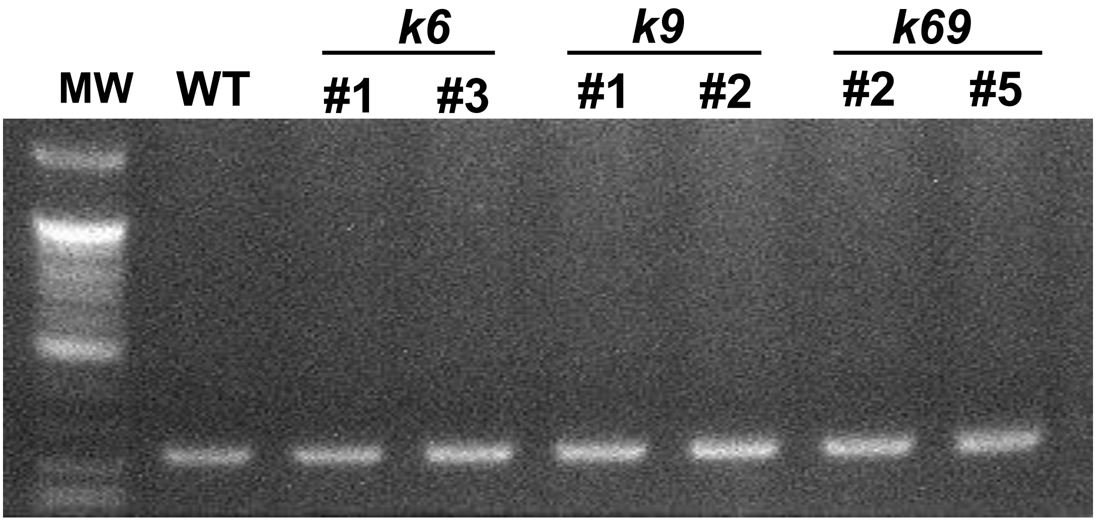

***rack1* qRT -Primers**

Figure S4. Polypeptide composition of thylakoid membranes. Image of two of the Western blot used for Immunotitration of thylakoid proteins in Fig. 2b. Specific antibodies against PSAA, CP43, LHCII and LHCA were used on cellulose on lanes loaded with 2, 1, 0.5 and 0.25 µg of Chls. On each gel wild-type (Wt) thylakoids were loaded in order to normalize the data.

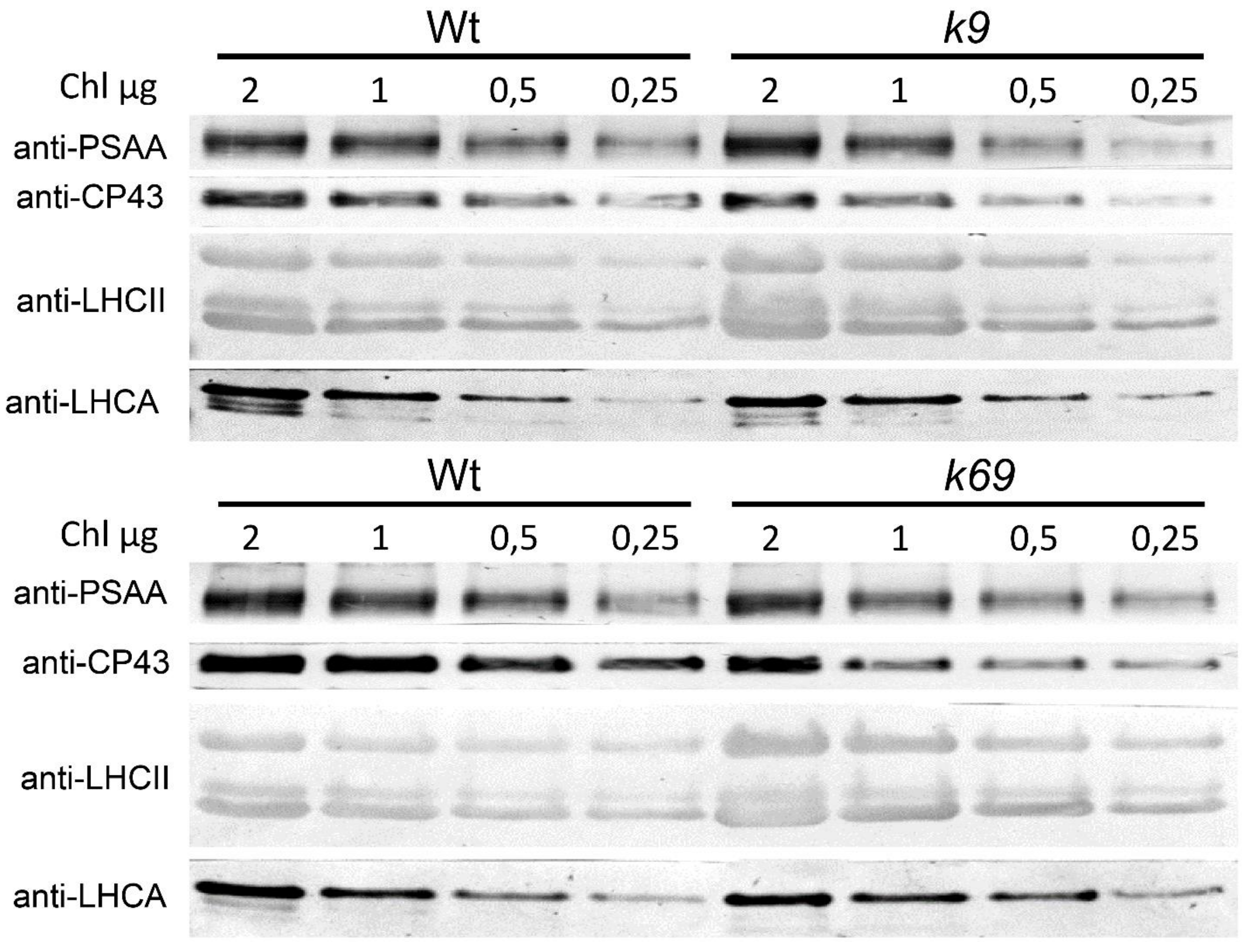

Figure S5. Densitometric analysis of sucrose gradients. Sucrose gradient loaded with solubilized thylakoids were analysed by densitometric analysis with GelPro extracting on green channel. Densitometric results are reported as optical density (OD) normalized to the total green of each gradient.

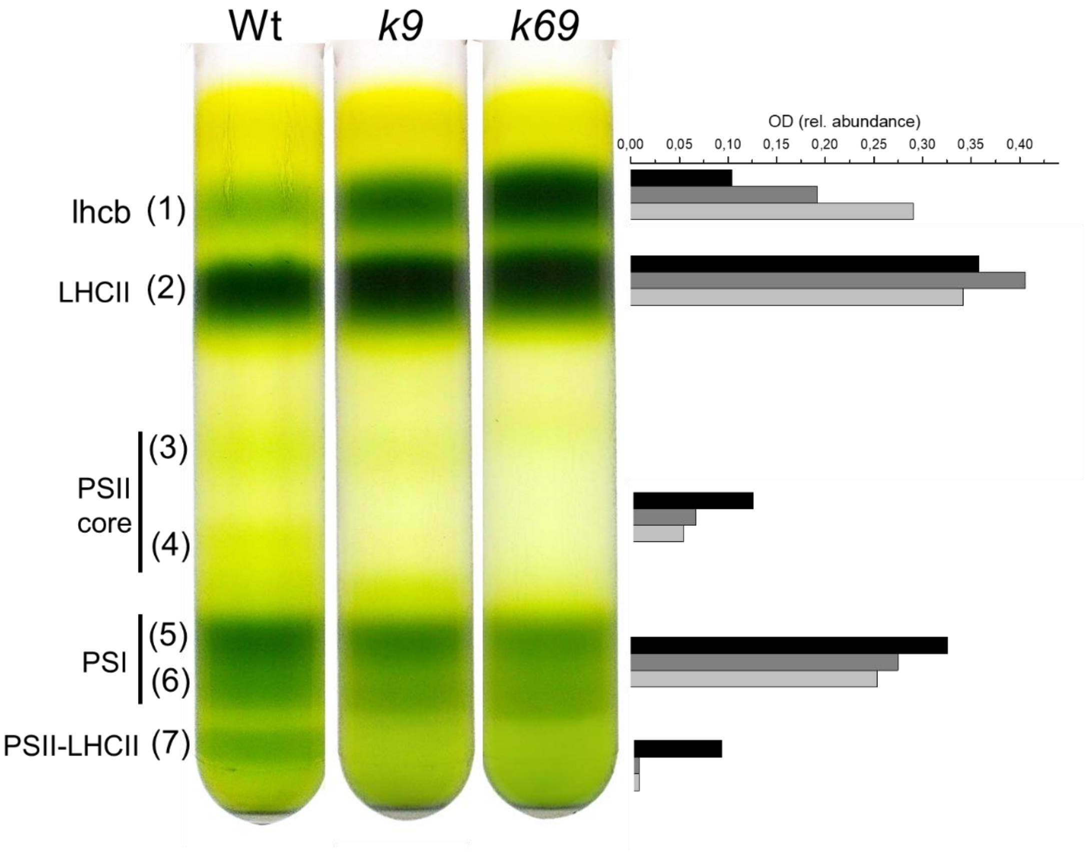

Figure S6. Functional PSII antenna size. Variable Chl fluorescence was induced with a weak red light of 11 μmol photons m^{-2} s^{-1}, on dark-adapted cells (about $2 \cdot 10^6$ cells/ml) in HS medium supplemented with 50 μM DCMU. The trace for wild-type (Wt, black), k9 (grey) and k69 (light grey) are the average of 40 curve for each genotype from four different experiments. The reciprocal of time corresponding to two-thirds of the fluorescence rise ($1/\tau_{2/3}$) is as a measure of the PSII functional antenna size and it is shown in the inset and in Table 1 normalized to the WT case, which was set to 100. Data are expressed as mean ± SD. Values that are significantly different (Student's t-test, P < 0.05) from the wild-type (WT) are marked with an asterisk (*). Date that are significantly different between k6 and k69 are marked with a circle (°).

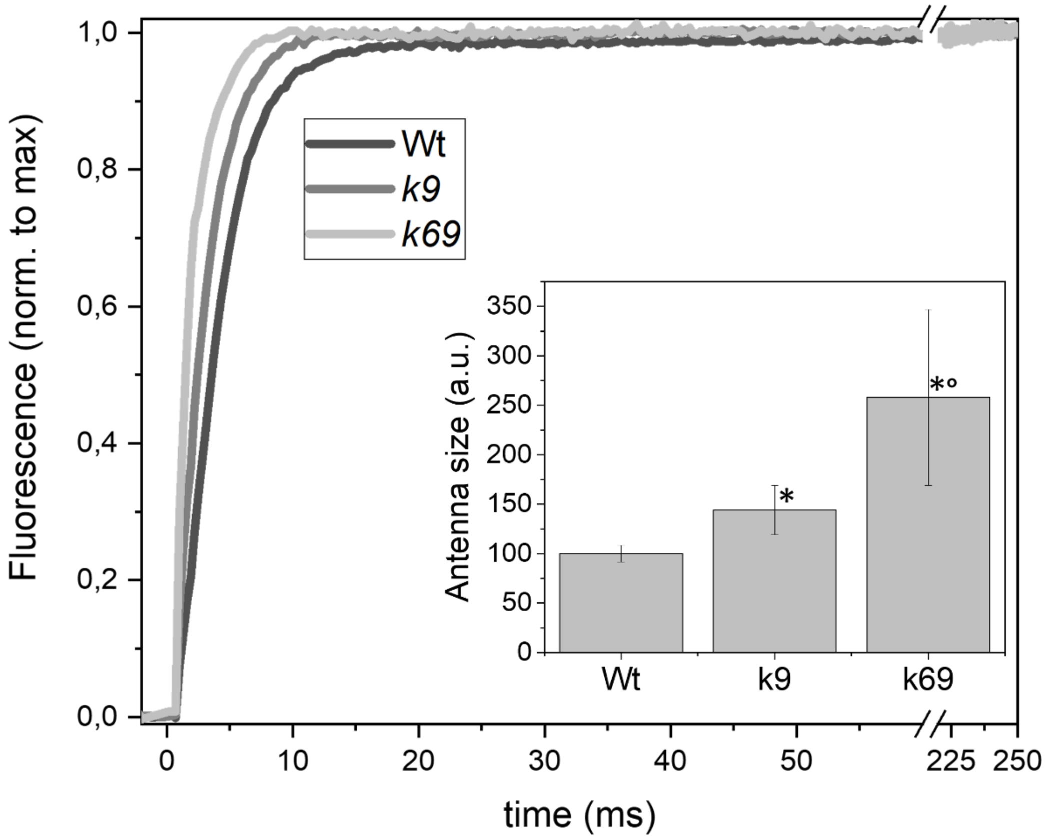

Figure S7. Western blot analysis of STT7 enzyme in Wt and mutant strains. Western blot were performed on STT7 kinase and CP43 used as loading control.

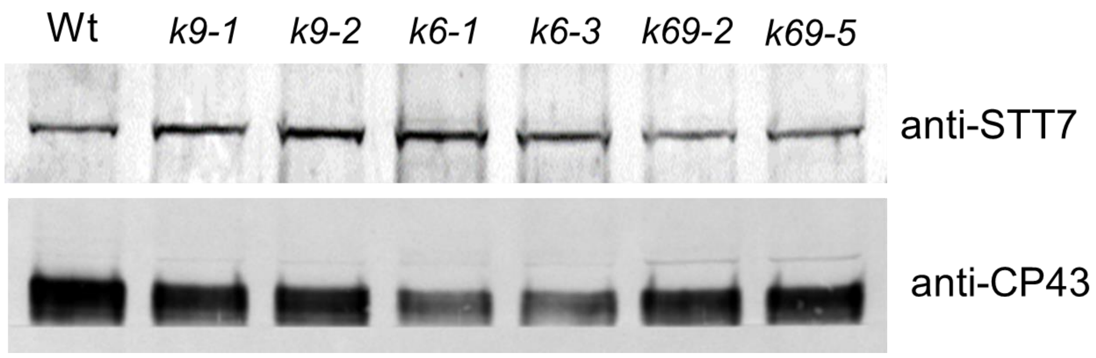

Figure S8. Quantification of minimal and maximum Chl fluorescence. The same amount of dark-adapted wild-type (Wt), k9 and k69 cells ($2 \cdot 10^6$ cells/ml) was excited with same PAM light setting and minimal (F_0) and maximal (F_m) were recorded. After the measure Chl were extracted and quantified from all the sample. F_0 and F_m were normalized to cells (**a,b** for F_0 and F_m respectively) and Chl content (**c,d** for F_0 and F_m respectively). Data are expressed as mean ± SD. Values that are significantly different (Student's t-test, $P < 0.05$) from the Wt are marked with an asterisk (*).

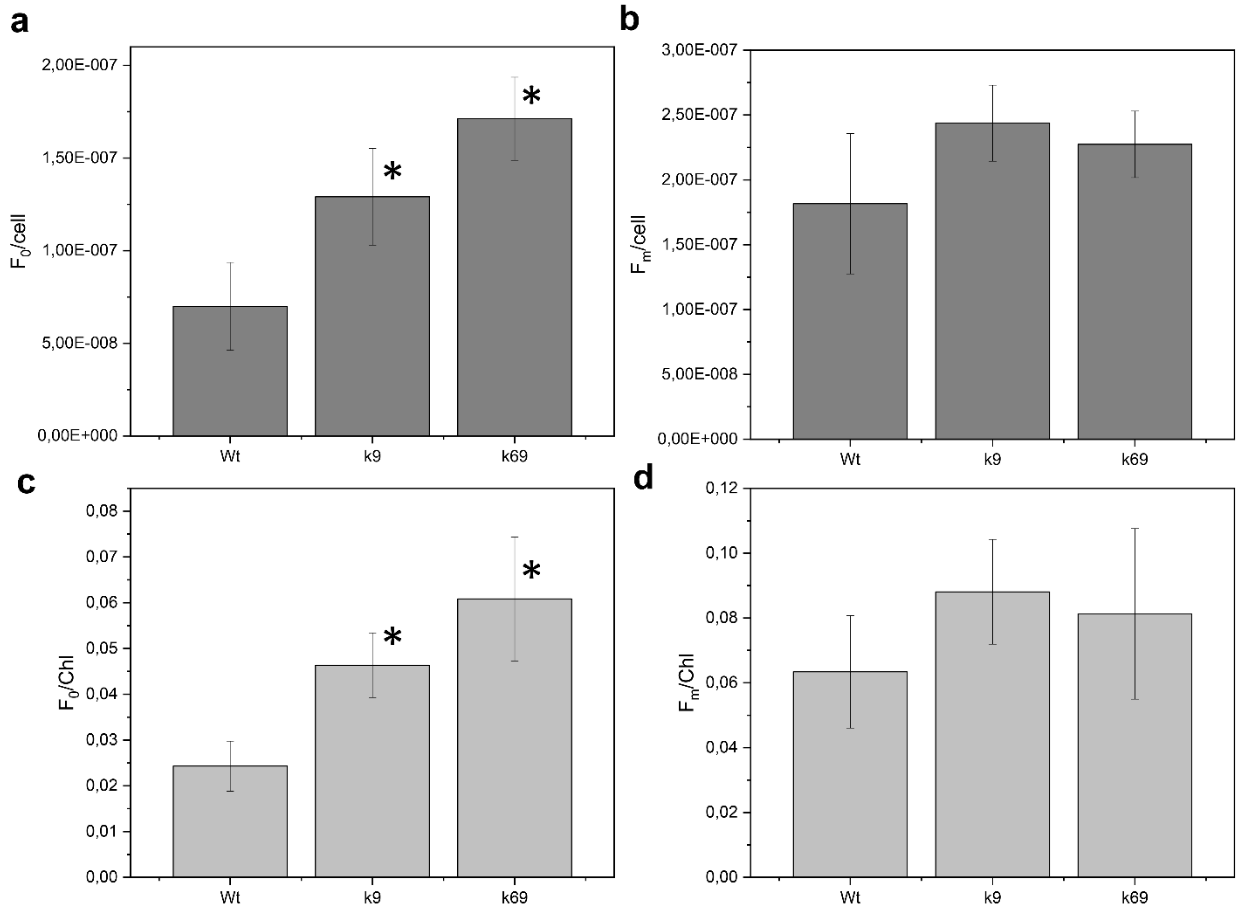

Figure S9. NPQ chlorophyll fluorescence. Example of fluorescence traces from wild-type (Wt black), k9 (grey) and k69 (light grey) obtained from NPQ measure using actinic light of 1200 µmol photons m^{-2} s^{-1}. Traces are vertically shifted to the same value of F_m.

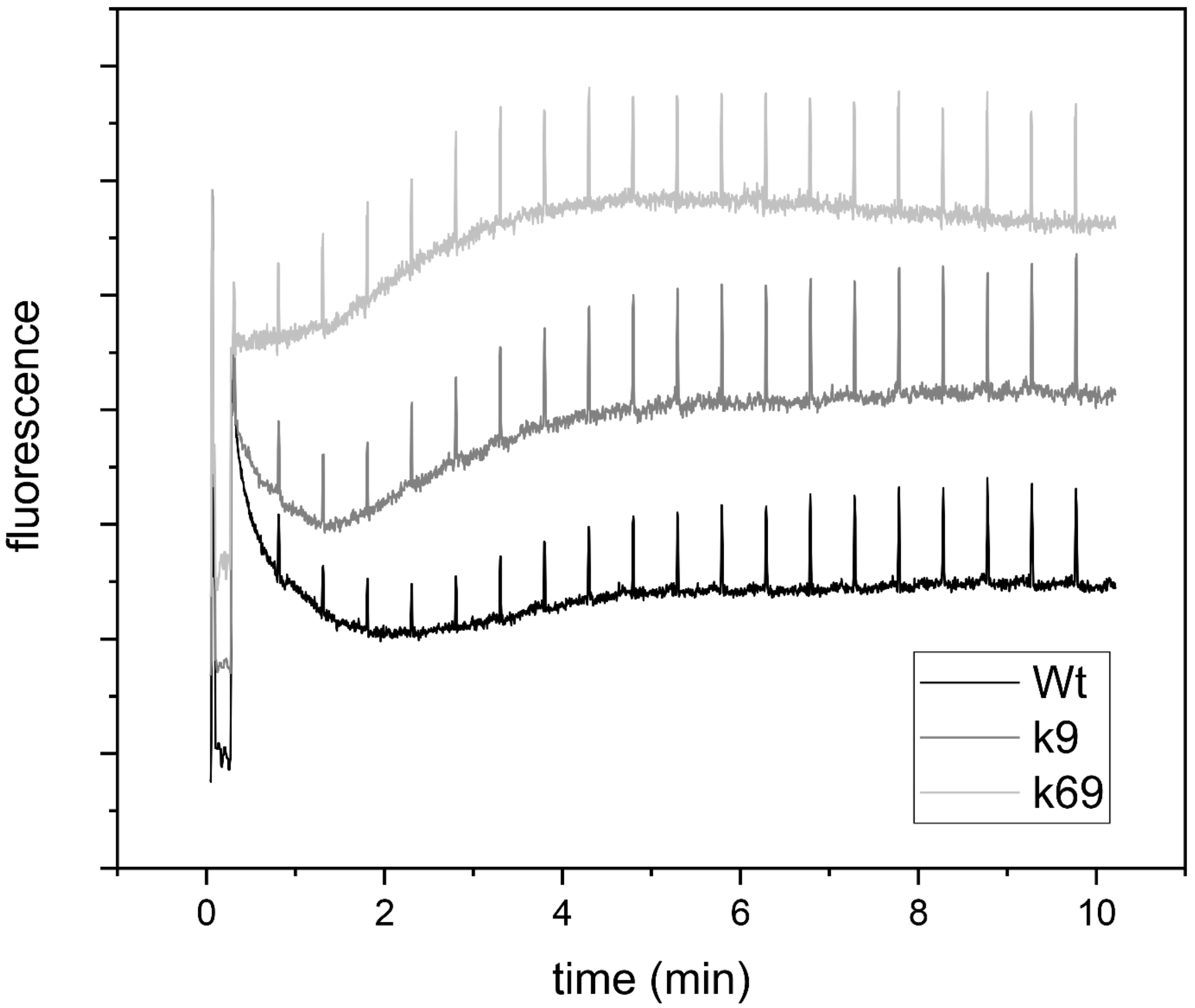

Figure S10. LHCSR quantification. Image of two of the Western blot used for immunotitration of thylakoid proteins in Fig. 5a. Specific antibodies against LHCSR3 and LHCSR1 were used on lanes loaded with 2, 1, 0,5 and 0,25 µg of Chl. On each gel wild-type (Wt) thylakoids were loaded in order to normalize the data. *npq4 lhcsr1* thylakoid (1 µg of Chl) was loaded as negative control.

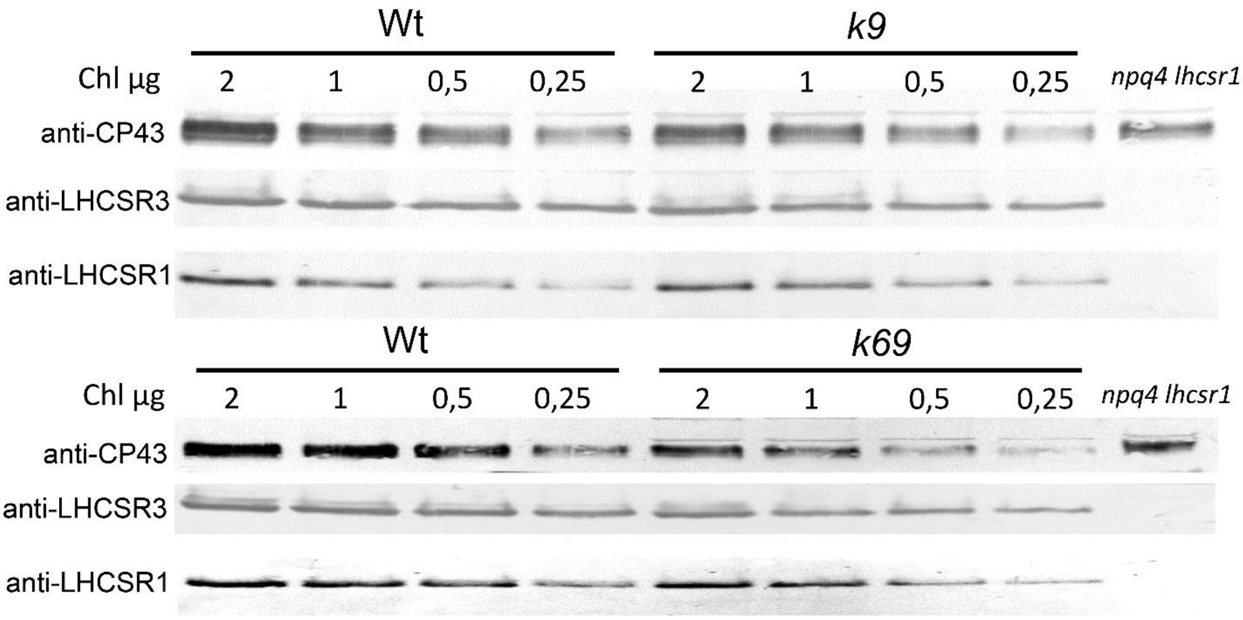

Figure S11. 77K fluorescence of WT and mutant strains. Low temperature fluorescence emission spectra of dark-adapted (dark) or light treated (light) wild-type (a,b), *k9* (c,d), *k69* (e,f) and *npq4 lhcsr1*(g,h) cells, shown if figure 7, were reconstructed by spectral deconvolution with Gaussians. Cumulative fit results are reported in red.

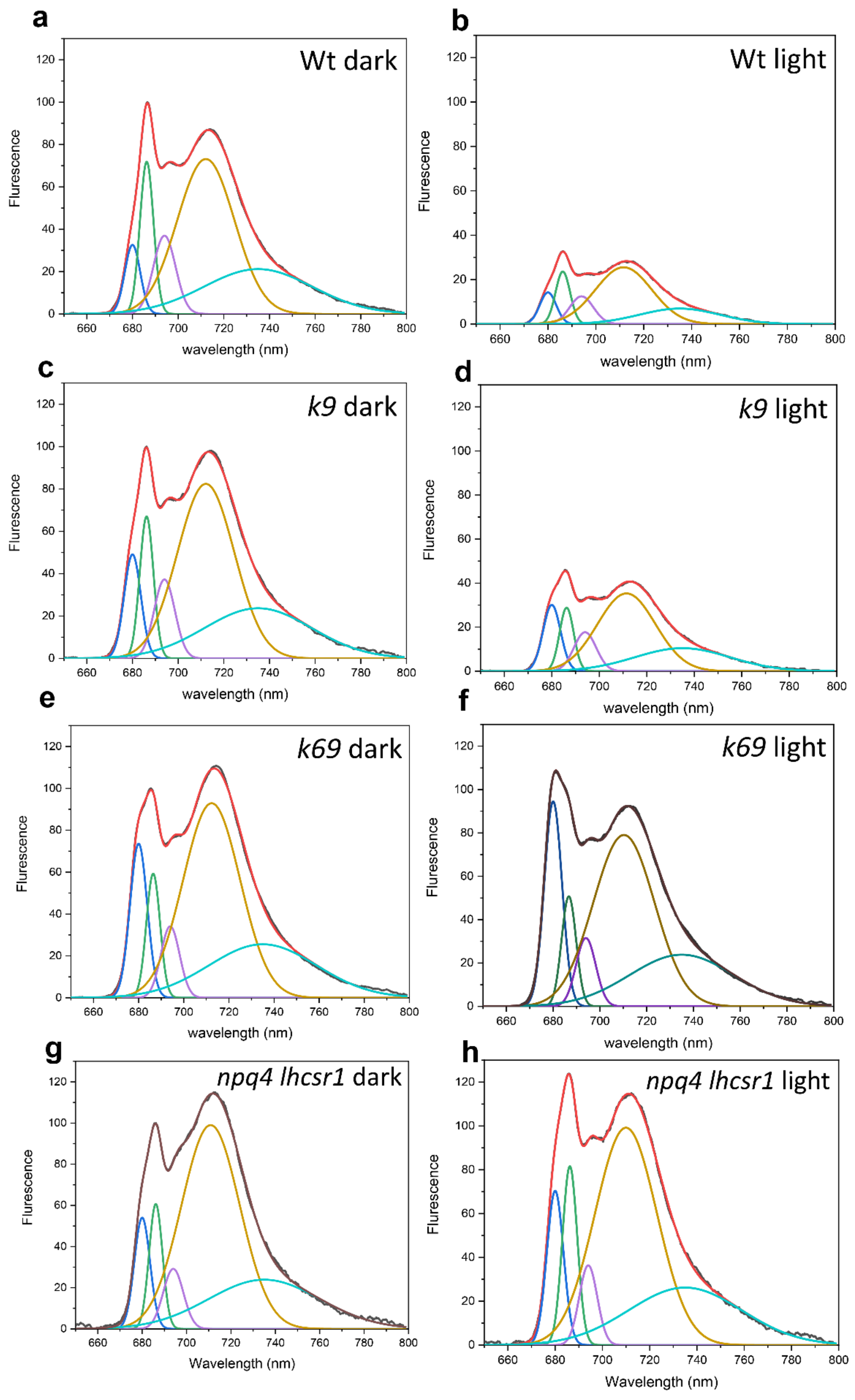

Table S1. Photosynthesis and respiration rates

	Wt	*k9*	*k69*
**Respiration in the dark (O_2 mmol cell^{-1} min^{-1})**	1.11 ± 0.25	1.10 ± 0.05	1.06 ± 0.21
**Pmax (O_2 nmol ug chl^{-1} min^{-1})**	10.47 ± 1.68	7.51 ± 0.24*	6.39 ± 1.32*
**Half-saturation intensity (µmol m^{-2} s^{-1})**	233 ± 23	247 ± 16	281 ± 21*°
**slope of linear increase (O_2 mmol cell^{-1} µmol m^{2})**	1.32 ± 0.18	0.78 ± 0.18*	0.72 ± 0.30*

Parameters extrapolated from oxygen light saturation curves shown in Fig. 3d. Data are expressed as mean ± SD. (n >3). Values that are significantly different (Student's t-test, $P < 0.05$) from Wt are marked with an asterisk (*). Date that are significantly different between k6 and k69 are marked with a circle (°).

Table S2. Pigment content of cell acclimated to 500 µmol photons m^{-2} s^{-1}.

				car/100 chl					
	pg chl/cell	chl a/b	chl/car	neo	viola	anth	lute	zea	b car
Wt	1.28 ± 0.28	2.70 ± 0.07	2.13 ± 0.10	4.69 ± 0.21	4.07 ± 0.82	2.35 ± 0.41	24.22 ± 0.82	2.64 ± 1.09	8.97 ± 0.20
k9	1.25 ± 0.16	2.55 ± 0.12	2.19 ± 0.14	4.23 ± 0.14*	3.96 ± 0.52	2.71 ± 0.03	23.90 ± 0.03	2.53 ± 0.71	7.92 ± 0.17*
k69	0.98 ± 0.16	2.48 ± 0.15*	2.13 ± 0.14	4.65 ± 0.32	4.13 ± 0.23	1.69 ± 0.35*°	25.84 ± 0.90*°	1.71 ± 0.36	8.78 ± 0.97

Single carotenoids values were normalized to 100 chlororophylls. Data are expressed as mean ± SD. (n = 4). Values that are significantly different (Student's t-test, $P < 0.05$) from the wild-type (Wt) are marked with an asterisk (*). Date that are significantly different between *k6* and *k69* are marked with a circle (°). Chl: chlorophylls; car: carotenoids; neo: neoxanthin; viola: violaxanthin; anth: anteraxanthin; lute: lutein; zea: zeaxanthin; β-car: β-carotene.

Table S3. Fv/Fm of cell acclimated to 500 µmol photons m^{-2} s^{-1}.

	**Wt**	***k9***	***k69***
**Fv/Fm**	0.67 ± 0.06	0.54 ± 0.06	0.46 ± 0.07

Photosystem II maximum quantum yield (Fv/Fm) measured in Wt and *k9* and *k69* mutant acclimated to high light (500 µmol photons m^{-2} s^{-1}). Data are expressed as mean ± SD. (n >3).
